# Species and Phenotypic Distribution Models Reveal Population Differentiation in Ethiopian Indigenous Chickens

**DOI:** 10.3389/fgene.2021.723360

**Published:** 2021-09-08

**Authors:** Fasil Getachew Kebede, Hans Komen, Tadelle Dessie, Setegn Worku Alemu, Olivier Hanotte, John W. M. Bastiaansen

**Affiliations:** ^1^Animal Breeding and Genomics, Wageningen University & Research, Wageningen, Netherlands; ^2^International Livestock Research Institute, Addis Ababa, Ethiopia; ^3^Cells, Organism and Molecular Genetics, School of Life Sciences, University of Nottingham, Nottingham, United Kingdom

**Keywords:** chickens, local adaptation, niche and agroecology, species distribution models (SDMs), phenotypic distribution models (PDMs), phenotypic differentiation, breeds and ecotypes, poultry genetics and breeding

## Abstract

Smallholder poultry production dominated by indigenous chickens is an important source of livelihoods for most rural households in Ethiopia. The long history of domestication and the presence of diverse agroecologies in Ethiopia create unique opportunities to study the effect of environmental selective pressures. Species distribution models (SDMs) and Phenotypic distribution models (PDMs) can be applied to investigate the relationship between environmental variation and phenotypic differentiation in wild animals and domestic populations. In the present study we used SDMs and PDMs to detect environmental variables related with habitat suitability and phenotypic differentiation among nondescript Ethiopian indigenous chicken populations. 34 environmental variables (climatic, soil, and vegetation) and 19 quantitative traits were analyzed for 513 adult chickens from 26 populations. To have high variation in the dataset for phenotypic and ecological parameters, animals were sampled from four spatial gradients (each represented by six to seven populations), located in different climatic zones and geographies. Three different ecotypes are proposed based on correlation test between habitat suitability maps and phenotypic clustering of sample populations. These specific ecotypes show phenotypic differentiation, likely in response to environmental selective pressures. Nine environmental variables with the highest contribution to habitat suitability are identified. The relationship between quantitative traits and a few of the environmental variables associated with habitat suitability is non-linear. Our results highlight the benefits of integrating species and phenotypic distribution modeling approaches in characterization of livestock populations, delineation of suitable habitats for specific breeds, and understanding of the relationship between ecological variables and quantitative traits, and underlying evolutionary processes.

## Introduction

Smallholder farmers in Africa keep scavenging poultry as a source of affordable animal protein and a means of income. The sustainability of this type of poultry production in tropical low-and medium-input systems depends on the availability of adaptive genotypes that can produce and thrive under adverse conditions such as climatic extremes, high prevalence of tropical diseases and parasites, and periodic feed shortage. The presence of selective pressures in these environments has led to adaptation of indigenous chicken populations to production constraints (Bettridge et al., 2018).

Local adaptation refers to local individuals having higher fitness in their environment than individuals from elsewhere (Williams, 1966). Environmental heterogeneity is known to be one of the main drivers of within species diversity and local adaptation (Darwin et al., 1858). Understanding the drivers of local adaptation provides essential information for designing research and development programs aiming at improving productivity while retaining resilience. A starting point in genetic improvement of the existing local chicken populations or in considering the introduction of new genotypes is to understand how the environment is driving local adaptation (Bettridge et al., 2018). This knowledge would allow breeding of indigenous ecotypes that are more productive under village conditions while retaining locally acceptable morphological and adaptive traits (Dana et al., 2010; Muchadeyi and Dzomba, 2017; Birhanu et al., 2021).

Present day African chickens are a result of an intricate interplay between domestication and natural selection. Ethiopia is an ecological microcosm of Africa, with a rich geomorphology, where people closely interacted with the environment and practiced agriculture for millennia. Because of its cultural diversity, geographical position, complex topography, and varying climatic patterns, the country harbors rich domestic animal biodiversity. The earliest osteological evidence of domestic chicken in Africa (921–801 BCE) was recovered from Ethiopia (Woldekiros and D’Andrea, 2017). The geomorphological landscape of the country is characterized by wide range of elevation (from –155 m to 4,620 m.a.s.l.) and diverse climate (Billi, 2015).

Recent technological advances in remote sensing and GIS, increased availability of environmental data, and improved computational power facilitate the understanding of the selective forces associated with local adaptation. Species distribution models (SDMs), implemented in MaxEnt (Phillips et al., 2006) and similar software, predict distribution of a species based on presence-only data, estimate the contribution of environmental variables, and help identify suitable habitats in current and future environments. Gheyas et al. (2021) and Lozano-Jaramillo M. et al. (2019) applied SDMs to produce suitability maps of Ethiopian chickens and identify important environmental variables associated with habitat suitability in chickens, without relating ecological differences with phenotypic variation among study populations. When used alone, SDMs treat a species as an evolutionarily homogenous entity and fail to consider possible population differences pertaining to local adaptation (Hampe, 2004). SDMs also make assumptions in their modeling approach (Wiens et al., 2009) which necessitate their combined use with additional approaches, such as phenotypic distribution models (PDMs).

Phenotypic distribution models use associations between phenotypes and environmental variables to map the phenotypes of populations within that species’ distribution (Michel et al., 2017). These phenotype-environment associations, are well documented for natural populations of several wild plant and animal species (Bergmann, 1848; Clausen et al., 1940; Mayr, 1942; Cain and Sheppard, 1954; Langerhans, 2008; Phillimore et al., 2010; Maloney et al., 2012; Michel et al., 2017; Smith et al., 2017) and can be applicable to predict phenotype distribution among domestic animals.

Phenotypic differentiation represents the fraction of phenotypic variance between populations over the total phenotypic variance and helps understand evolutionary processes shaping populations (Storz, 2002; Leinonen et al., 2006; Schmid and Guillaume, 2017). With the exception of Lozano-Jaramillo A. et al. (2019) who applied PDMs to predict performance of improved chicken strains, distribution models have seldom been applied in indigenous livestock to identify environmental factors associated with phenotypic differentiation and to define their ecotypes. In contrast to introduced strains which have been subjected to intense artificial selection in a relatively short period of time, indigenous populations have been exposed to natural selection over multiple generations which permits a better understanding of evolutionary processes. Even with natural populations of animals, correlation between a phenotype and environment could be spurious if PDM are used on their own (Etterson and Shaw, 2001; Michel, 2011; Michel et al., 2017) and this requires their combination with additional analytical approaches, such as SDMs.

To overcome possible limitations in the use of SDMs in domesticated species like livestock, where humans may have interfered in the geographic distribution of the study species, we have taken corrective measures in our study design. Our sampling strategy was elaborate enough to ensure environments potentially habitable by chickens are included in sufficient sample size, while those uninhabitable are excluded in the sampling frame. We targeted random mating, nondescript indigenous chicken populations from separate livestock market-sheds, clustered along environmental gradients, to maximize ecological and phenotypic variation between sample populations. We followed a novel approach integrating SDMs with PDMs through generalized additive models (GAMs) to identify the most important environmental variables contributing to habitat suitability and evaluate their relationships with phenotypic differentiation among Ethiopian indigenous chicken populations.

## Materials and Methods

### Sampling Strategy

**FIGURE 1:**
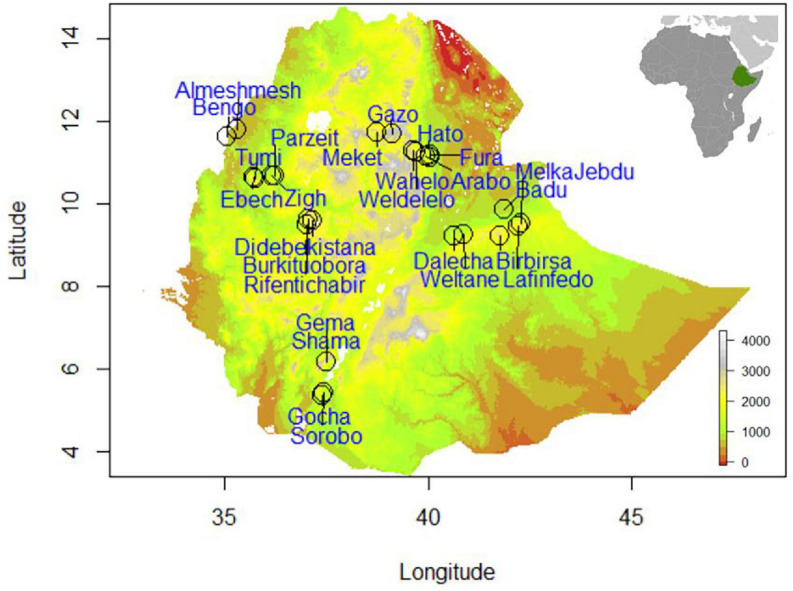
Topographic map of Ethiopia depicting the 26 indigenous chicken sample populations.

A hybrid strategy, maximizing both environmental and geographical representativeness of sampling sites, increases statistical power by reducing false discovery rates caused by demographic processes and confounding effects (De Mita et al., 2013; Lotterhos and Whitlock, 2015; Selmoni et al., 2020). We used a hybrid sampling strategy that covered the target area, ensuring high environmental variability, wide geographic distributions, and considering the demographic and biotic processes influencing the Ethiopian indigenous chicken populations (Figure 1). Chickens were sampled from four spatial gradients with a minimum distance between gradients of 500 km. Each gradient comprised three environmental clusters, primarily delineated based on elevation (400–1,800; 1,800–2,400; and 2,400–3,500 m.a.s.l.).

While we did not consider administrative regions in Ethiopia in our sampling strategy, we would like to describe the four regions covered in the present study (Amhara, Oromia, Benishangul-Gumuz, and Southern regions) to give a brief view of the geographic landscape. Gradient-I stretched from the Rift valley lowlands of northeastern Ethiopia (McConnell, 1967), along the territories of Afar region to the highlands of Wollo province within Amhara region. Gradient-II, starts from the Rift valley lowlands in central Ethiopia, crosses the highlands of Hararghe, including Mount Gara Muleta, and stretches to eastern Ethiopia within Oromia region. Gradient-III stretches from the highlands of northwestern Ethiopia and goes down to the lowlands along the Ethiopian–Sudanese border within Benishangul-Gumuz region. Gradient-IV spans from the highlands of western Ethiopia in Oromia region to the lowlands along the Ethiopian–Kenyan border in Southern region. Areas around the national borders of Ethiopia have low elevation, which gradually culminate to highland plateaus in the center of the country creating a striking contrast in agroecology (Ethiopian Mapping Authority, 1988). Geographic coordinates and phenotypic measurements were not taken from areas which are not habitable by chickens because of their unconducive environments (below 400 and above 3,500 m.a.s.l.).

**FIGURE 2:**
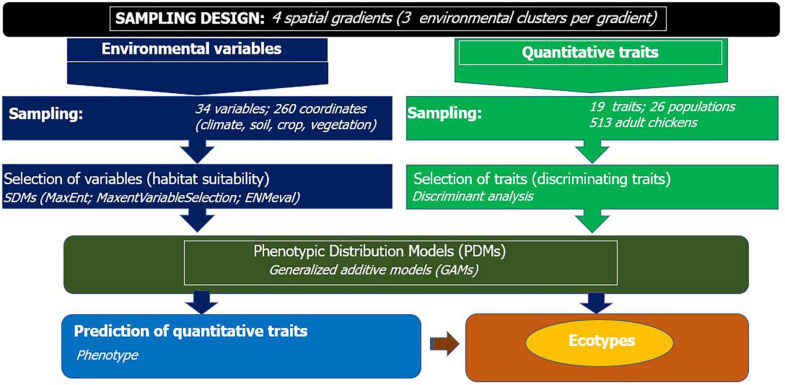
Workflow for integrated species and phenotypic distribution modeling to detect population differentiation and define ecotypes of indigenous chickens.

We made sure that clusters within a gradient were distant by at least 100 km and the target chicken populations were sampled from households which visit isolated, i.e., not connected livestock market-sheds. The concept of market-shed refers to a geographic area, where households therein are in sufficient proximity to exchange their animals in various ways (e.g., sale, gift), most commonly traveling on foot. Each cluster along the spatial gradient constituted two to three populations. A total of 26 populations were sampled (Figure 1 and Supplementary Table 1). The sampling frame is spatially evenly spread to capture high inter- and intrapopulation environmental and quantitative trait variability. The research design integrating SDMs and PDMs is presented in Figure 2.

The sample locations in our study covered 13 out of the total of 18 agroecological zones (MoA, 1998; Tadesse et al., 2006) in Ethiopia. Agroecological zonation utilizes biophysical attributes of soil, terrain, and climate to organize land-use types or production systems into relatively homogenous units (Hurni, 1998). The five agroecologies that were not covered, are areas where chickens have either not been reared due to extreme climates, cannot be kept at all (e.g., water bodies, undisturbed forests), or have only been recently introduced.

### Environmental Data

A total of 34 environmental variables were selected for their potential effects on chicken adaptive evolution. Data on these variables was extracted from online databases (Supplementary Table 2). The environmental data included bioclimatic (*n* = 24), vegetation (*n* = 2), and soil (*n* = 8) variables. Values for bioclimatic variables (temperature, precipitation, solar radiation, and water vapor pressure) in different seasons were obtained from WorldClim database^1^ at a spatial resolution of 30 s (∼1 km^2^; Fick and Hijmans, 2017) based on mean values of 30 years (1970–2000). Cropland extent at 30-m resolution was attained from Global Food Security Analysis-Support Data (Xiong et al., 2017). The SoilGrids system at 250 m resolution with standard numeric soil properties (organic carbon, bulk density, cation exchange capacity, pH, soil texture fractions at 15–30 cm depth was accessed from ISRIC database; Hengl et al., 2015, 2017). In addition to the 34 environmental variables elevation data was downloaded from DIVA-GIS^2^ (Hijmans et al., 2001; Farr et al., 2007) at a spatial resolution of 30 s (∼1 km^2^).

### Species Distribution Models

Species distribution models (also called niche, envelope, or bioclimatic models) associate georeferenced observations of a biotic response variable – typically species occurrence or abundance – with multiple environmental predictors using a broad array of statistical learning methods to describe species’ niches (Elith and Leathwick, 2009; Franklin, 2010; Elith and Franklin, 2013; MacKenzie et al., 2017). MaxEnt is a general-purpose machine learning algorithm developed to model species distributions from presence-only records (Phillips et al., 2006).

For every population, a single geographic coordinate was taken at the center of the village during sampling of chickens. Coordinates from nine additional grids (1.44 km^2^), covering a total of 12.96 km^2^, were then drawn around a recorded location and extracted using Google Earth Pro v 7.3.2 to ensure high representation of environmental variability affecting the population (Gheyas et al., 2021). This way the total number of “presence” or “occurrence” points used in SDMs for the 26 sample populations comprised 260 coordinates. Different R software packages: “*sp*” (Pebesma et al., 2012), “*raster*” (Hijmans et al., 2015), “*rgdal*” (Bivand et al., 2015), “*maptools*” (Bivand et al., 2021), “*rgeos”* (Bivand et al., 2017), and “*dismo*” (Hijmans et al., 2017), were used to extract, read, and visualize geospatial data. Dimension and extent of the grids were corrected and homogenized for 1 km^2^ based on the WGS84 geodetic reference system (Decker, 1986).

### Selection of Environmental Variables

To constrain model complexity and increase the performance of SDMs, the highest contributing set of uncorrelated environmental variables were identified and Maxent’s regularization multiplier was fine-tuned using the R package “*MaxentVariableSelection*” (Jueterbock et al., 2016). The predictive performance of the most important environmental variables was measured using test gain in MaxEnt v.3.4.1 (Phillips et al., 2006; Phillips and Dudík, 2008).

### Configuration of Model Parameters

Species-specific tuning of model parameters can improve the performance of MaxEnt model compared to the default settings (Elith et al., 2011; Radosavljevic and Anderson, 2014). The large set of feature types was subsequently reduced to the optimal subset to improve model fit and the optimum regularization multiplier for model training was identified by the R package “*ENMeval*” (Muscarella et al., 2014) by using spatial blocks method (Radosavljevic and Anderson, 2014). Regularization refers to smoothing the model, making it more regular, to avoid fitting too complex a model. It is a common approach in model selection and penalizes coefficients (the betas) to values that allow both accurate prediction and generality (Tibshirani, 1996; Elith et al., 2011).

Species’ responses to environmental covariates or independent variables (e.g., temperature, elevation) tends to be complex and usually requires fitting of non-linear functions (Austin, 2002). In machine learning algorithms this is achieved by applying transformations of the original covariates into features. MaxEnt currently has six feature classes: linear, product, quadratic, hinge, threshold, and categorical (Elith et al., 2011). We built models with regularization multiplier values ranging from 0.5 to 4.0 (increments of 0.5) and with six different feature combinations (H, LQH, HQP, HQC, LQHP, LQHPT; where L, linear; Q, quadratic; H, hinge; P, product; and T, threshold); this resulted in 48 individual model runs. The parameter configuration with the lowest delta AICc value was chosen to run the model (Supplementary Table 3). To reduce the influence of sampling bias, we included a bias file (Phillips et al., 2009) and preferentially sampled pseudo background points from areas near our presence points based on kernel density function (Venables and Ripley, 2002).

### Tests of Niche Similarity

A niche is a description of the conditions in which a species maintains a viable population. Populations in a species that are adapted to a specific local habitat or niche show genetically induced phenotypic differences in response to environmental selective pressures and are regarded as “ecotypes” (Müntzing, 1971; Knüpffer et al., 2003). Niche similarity between one or more pairs of populations was measured according to Warren et al. (2008). Raster files (.ASCII) of predicted habitat suitability produced by MaxEnt in logistic output (no probability and complete probability of presence designated by 0 and 1, respectively) were used as inputs to perform correlation test by ENMTools (Warren et al., 2010). Correlation tests were used to cluster sampling sites on the selected environmental variables and build dendrogram through hierarchical clustering with R package *cluster* (Maechler et al., 2013). The grouping of sampling locations into environmental niches was based on “Euclidean” distance. Different clustering methods (Ward’s minimum variance method, complete linkage, average linkage, and single linkage) were compared. Visualization of the cluster memberships of locations of populations based on niche similarity, measured by correlations tests on the most important environmental variables, was accomplished using the R package *factoextra* (Kassambara and Mundt, 2017).

### Quantitative Trait Data

A total of 19 phenotypic traits (Supplementary Table 4), selected for their potential role in adaptation in chicken based on available literature, were measured on 513 adult chickens (380 hens and 133 cocks) from the 26 nondescript indigenous chicken sample populations. We had three environmental clusters (lowland: 400–1,800; midaltitude: 1,800–2,400; and highland: 2,400–3,500 m.a.s.l.) stretching across each of the four elevational gradients in this study. A total of 12 environmental clusters from the four elevational gradients were included. Each environmental cluster is represented by two randomly selected chicken populations, except in two instances where we took three populations. A population refers to the total number of nondescript indigenous chickens available in an administrative village. Adult chickens were selected randomly for phenotyping through transect walk across villages. This method entailed walking along a defined path (transect) across a village and sampling one chicken from each farming household until a total of 15 hens and five cocks were measured.

The age of the chickens was estimated by interviewing owners to confirm that females were in their second clutch (7–8 months-of-age) and males were above 12 months-of-age. The researchers also visually appraised cocks (roosters) for presence of well-developed spurs. One chicken was sampled per household. Under rare circumstances (*n* = 9 households), two chickens were sampled per household when farmers proved their animals have no family relationship.

Live bodyweight of individuals was taken in the morning on fasting chickens. Accurate morphological measurements were made by using different tools (digital balance, measuring tapes, and image processing software) Supplementary Table 4. The pictures of individual chickens taken in a sheltered environment to achieve appropriate resolution were digitally analyzed using *ImageJ* (Rasband, 1997). To reduce systematic error, the same operator measured all chickens, which were held in the same position by a technician. A steel ruler was placed in the background of every picture as a distance reference.

### Selection of Quantitative Traits

A multivariate test of differences between populations with stepwise selection (Klecka et al., 1980) was performed through linear discriminant function analysis (SAS, 2002) to identify the traits which were most useful in classifying populations. Principal component analysis (PCA) was run with R “*stats*” package on quantitative trait data to see how much percent of the variation is explained by the first nine principal components (PCs).

### Clustering of Nondescript Chicken Populations Into Ecotypes

The 26 nondescript Ethiopian chicken populations sampled in this study are heterogenous in terms of qualitative traits (e.g., coat color, comb shape, and feather pattern) and quantitative traits. We used the most discriminant quantitative traits, which are most useful because of their variability, to group populations into ecotypes. We expect that populations of chickens within the same niche are affected by similar environmental variables and cluster into the same ecotype. The phenotypic values of these traits were analyzed by the average silhouette method to decide on the optimal number of clusters. The average silhouette method measures how well each experimental unit lies within its cluster and is less ambiguous than the elbow method to decide on the number of clusters (Rousseeuw, 1987; Kaufman and Rousseeuw, 2009).

Different hierarchical clustering methods (Ward’s minimum variance method, complete linkage, average linkage, and single linkage) were compared via R packages “*cluster*” (Maechler et al., 2013) and “*factoextra*” (Kassambara and Mundt, 2017) to make a valid comparison of population memberships between dendrograms produced on similarity of phenotypes. We used the same approach for clustering of environmental and phenotypic data to avoid any possible bias associated with the use of different tools.

### Phenotypic Distribution Models

While species can vary genetically and phenotypically across their range and populations can be locally adapted, SDMs assume that all populations respond homogenously to the range of environmental conditions experienced by the whole species (Bolnick et al., 2003; Atkins and Travis, 2010; Fitzpatrick and Keller, 2015; Hällfors et al., 2016). PDMs on the other hand, do capture the response of quantitative traits as a function of environmental conditions (Michel et al., 2017; Smith et al., 2017; Lozano-Jaramillo A. et al., 2019). We used PDMs to study variation within quantitative traits in response to the most important set of environmental variables identified by SDMs. The association of these environmental variables with habitat suitability were evaluated for their individual effect on each of the discriminating traits. The relationship between quantitative traits and environmental variables was expected to be non-linear (Zuur et al., 2007; Oddi et al., 2019). The assumptions of classical statistical approaches such as generalized linear models (GLM) are violated when responses are non-linear, variances change with predictors, or ecological processes operate at spatio-temporal scales (Zuur et al., 2009; Bolker et al., 2013).

Exploration of phenotypic and environmental data was initially carried out to understand their distribution, variance structure, and linearity or non-linearity of trend and to choose appropriate analytical methods. GAMs were selected because they are particularly useful for analyzing relationships explained by complicated shapes, such as hump-shaped curves (Crawley, 2012). The R package “*mgcv*” (Wood and Augustin, 2002) was used to fit GAMs (Hastie and Tibshirani, 1990). Model validation was made based on Akaike information criterion (AIC) values.

The response of each quantitative trait was predicted as a function of ecotype, niche, and the six SDM-selected environmental variables. The GAM included ecotypes and their respective niches as linear terms and the environmental covariates as smoothing parameters. The notation for the GAM smoothing in a Gaussian model is as follows (Hastie and Tibshirani, 1990; Wood and Augustin, 2002).

$$g\,\left(E\,\left(y_{i}\right)\right)=\alpha +\beta_{j}+\gamma_{m}+f_{k}\,\left(X_{k\,i}\right)\,\ldots ,$$

Where (*E*(*y*_*i*_)) is one of *n* observations of the response trait, *g* is the Gaussian distributed exponential family with identity link function, α is the intercept,β_*j*_ is a linear parameter for ecotype (1,2,3), γ_*m*_ is a linear parameter for environmental niches (1,2,3), *f*_*k*_ are the smoothing terms based on non-parametric predictor covariates *X*_*ki*_ (the shape of the predictor functions which will be fully determined by the data structure).

Estimation of smoothing parameters effects (environmental variables) was done by restricted maximum likelihood as random effects (Wiley and Wiley, 2019) with Gaussian process smooth (bs = “gp”) in the GAMs model (Wood, 2012).

Partial dependence plots (PDPs; Friedman, 2001) are the most popular approach for visualizing the effects of the predictor variables on the predicted outcome during supervised machine learning applications (Apley and Zhu, 2020). A PDP can show whether the relationship between the target and a feature is linear, monotonic, or more complex. PDPs exhibiting the effects of environmental factors with estimated *p*-value on a phenotype were produced by using the R package “*mgcViz*” (Fasiolo et al., 2020) at 95% confidence interval.

## Results

### Environmental Variables Contribute to Habitat Suitability

#### Optimum Model Parameters

**FIGURE 3:**
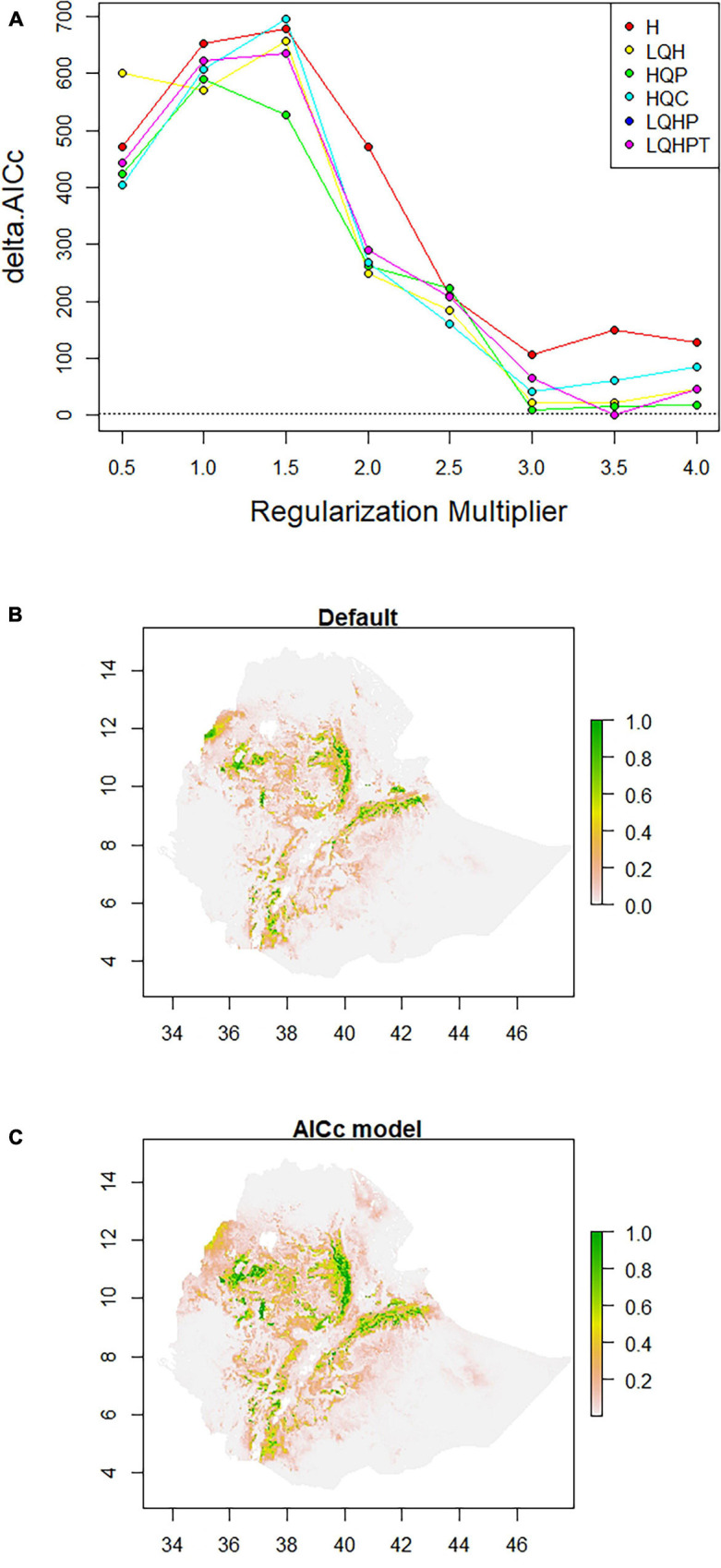
Model configuration and habitat suitability maps for Ethiopian indigenous chicken populations. **(A)** AICc values for analyzed feature combinations using different regularization-multipliers ranging from 0.5 to 4.0. Feature combinations include one or more of the following types: L, linear; Q, quadratic; H, hinge; P, product; and T, threshold. **(B)** Map produced using default settings of MaxEnt. **(C)** Map produced using optimum parameters (HQP features with regularization-multiplier = 3.0) identified by ENMeval.

ENMeval identified HQP (Hinge, Quadratic, and Product) features with regularization-multiplier = 3.0 as the best parameter combination. This had the lowest deltaAICc value and was chosen to produce suitability maps by MaxEnt (Figure 3A). Compared to the default (Figure 3B), the model fit with the optimum parameters predicted larger areas as most suitable for poultry production (Figure 3C). The areas least populated by chickens include the extreme lowlands (below 400 m.a.s.l.), with prohibitively high temperature, high solar radiation, low precipitation, and high relative humidity; and the extreme highlands (above 3,400 m.a.s.l), with prohibitively low temperatures. The extreme highlands are frosty and hence not habitable both by livestock and humans. Ethiopian lowland pastoral areas are affected by recurrent drought and have generally sparse livestock population (Tilahun and Schmidt, 2012). Agreement between the results of the present study and the census report (CSA, 2017) and other literature indicating the distribution of livestock (Tilahun and Schmidt, 2012) confirm that those areas in the country which are shown as least suitable in the habitat suitability maps produced by SDMs are indeed unsuitable for the study species. Sedentary systems in central Ethiopia have conducive environmental conditions for chickens while pastoral systems (hot, dry areas, with strong solar radiation) along the borders of the country do not (Getahun, 1978; Bayou and Assefa, 1989; CSA, 2017; Mirkena et al., 2018; Gebrechorkos et al., 2019). The choice of livestock species to rear is also culturally embedded over generations.

#### Most Contributing Environmental Variables

**FIGURE 4:**
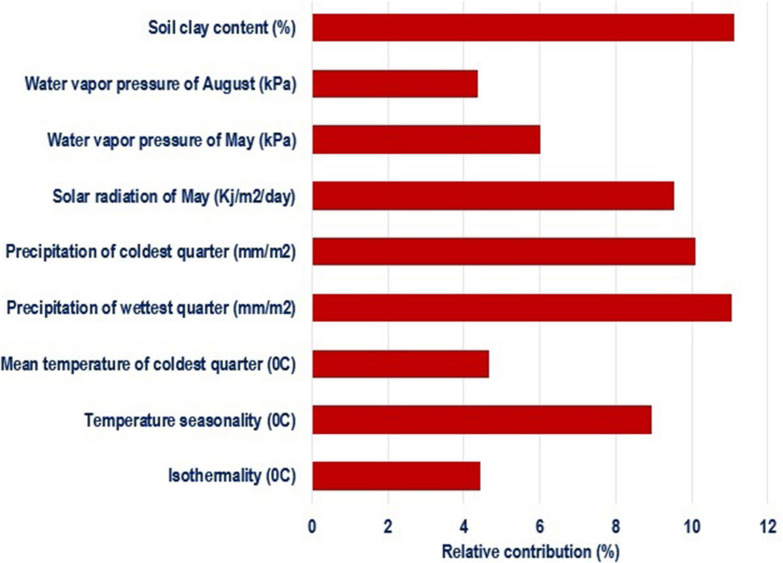
Environmental variables of importance and their percent contribution predicted by MaxentVariableSelection.

Species distribution models identified the most important environmental variables associated with distribution of chickens (Figure 4). Correlated variables (| *r*| > 0.6) and those with a relative contribution score below 4% were removed to restrict multicollinearity driven effects in projecting species ranges (Dormann et al., 2013; Brun et al., 2019). Out of 34 environmental variables, nine were retained as most important in determining habitat suitability and can be regarded as potential drivers of local adaptation in Ethiopian indigenous chickens. The first five variables with the highest contribution included soil clay content, precipitation of the warmest quarter, precipitation of the coldest quarter, and temperature seasonality.

**FIGURE 5:**
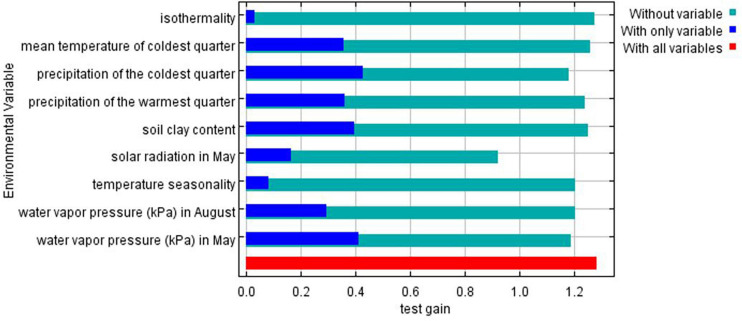
Gains of the variables in the Maxent model (Jackknife test) for Ethiopian indigenous chickens. Turquoise bars: model gain without corresponding variables; blue bars: model gain with only the corresponding variables; red bars: total gain using all the variables.

Jackknife test was run to compare the relative importance of the nine selected environmental variables (Figure 5). The test showed that precipitation of the coldest quarter and water vapor pressure in May have the highest gain when used in isolation, and therefore are the most useful variables for predicting the distribution of the species on occurrence data. On the other hand, the environmental variable that decreases gain the most when omitted is solar radiation in May, meaning it has the most important information that is not present in other variables.

### Distinct Niches Are Associated With Distinct Ecotypes

**FIGURE 6:**
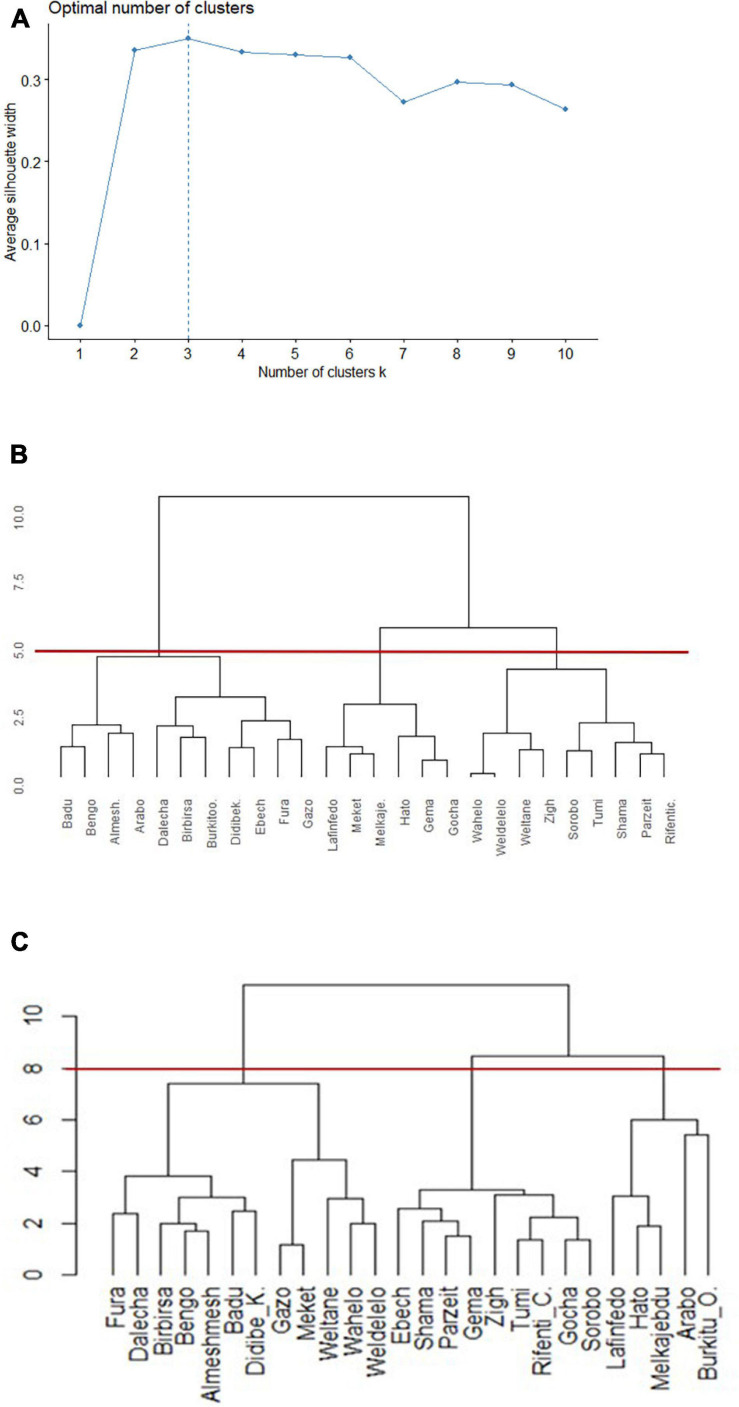
Dendrograms of clusters to group 26 Ethiopian indigenous chicken populations (hierarchical agglomerative clustering, Ward’s minimum variance method). **(A)** Plot of statistics for deciding appropriate number of clusters based on phenotype. **(B)** Dendrogram based on niche overlap statistic (*I*) between suitability maps. The red line at a cutoff value of 5.0 produces three niches. **(C)** Dendrogram based on the most discriminating phenotypes. The red line at a cutoff value of 8.0 produces three distinct ecotypes.

Populations of animals adapted to a specific environment or niche are regarded as ecotypes. Clustering of sample chicken populations into phenotypically homogenous groups and an overlap of the clustered populations with niche classification based on their respective environments was used as a basis to define ecotypes. The number of chicken ecotypes was determined through Silhouette method using phenotypic data (Figure 6A). The optimal cluster in the present study, the one that maximized the average silhouette from a range of possible *k* values, was *k* = 3. The same clustering method (Ward, 1963) was used to make a valid comparison of population memberships between dendrograms produced on similarity of niches (Figure 6B) and on similarity of phenotypes (Figure 6C).

Populations were clustered into three environmental niches based on correlation test (Figure 6B). Ward’s method had the strongest clustering structure for clustering on niche overlap (Ward = 0.89). The agglomerative coefficients for the other approaches (complete linkage = 0.78; average linkage (UPGMA) = 0.68; and single linkage = 0.36) was lower. At a cutoff value of 5.0, reading the plot from left to right, niche-I comprised 11 sampling locations, while niche-II and niche-III comprised six and nine locations, respectively.

### Variation in Quantitative Traits

Before classifying the 26 sample chicken populations into ecotypes through hierarchical clustering based on similarity for quantitative traits, we reduced the number of traits through discriminant analysis (Table 1). Out of 19 quantitative traits (Supplementary Table 4), eight (BL, WS, CL, CW, BW, EW, WW, and KL) had the highest discriminant function because of their high variation between populations. Except wattle width (*p* < 0.05), the remaining seven of these eight discriminant traits showed highly significant phenotypic variation (*p* < 0.0001 to *p* < 0.01) between female sample chicken populations. The GLM analysis combining data from both sexes revealed all the discriminating quantitative traits varied significantly between sexes (*p* < 0.0001) except for beak length (*p* = 0.1738). The partial *r*-square indicates body length (BL) had the highest discriminatory effect out of all traits retained in the models in both sexes. Only two quantitative traits (BL and BW) were found useful (*p* < 0.0001) for classifying male sample chicken populations. This might be related with their lower sample size or a different structure of morphological variation among male sample populations compared to females.

**TABLE 1:** Stepwise selection summary indicating most discriminating traits for adult male and female Ethiopian indigenous chicken sample populations.

Sex	Quantitative trait	Partial R-sq.	*F* value	Pr > F
Hens	BL	0.4761	13.51	<0.0001
	WS	0.2934	6.15	<0.0001
	CL	0.2274	4.34	<0.0001
	CW	0.1766	3.15	<0.0001
	BW	0.1741	3.08	<0.0002
	EW	0.1677	2.93	<0.0003
	WW	0.1184	1.63	<0.0214
	KL	0.1534	1.93	<0.0014
Cocks	BL	0.7756	14.52	<0.0001
	BW	0.4856	3.9	<0.0001

*BL = body length (mm); WS = wingspan (mm); CL = comb length (mm); BW = body weight(g); EW = earlobe width (mm); WW = wattle width (mm); and KL = beak length (mm).*

A subset of quantitative traits that best revealed the differences among chicken populations (Table 1) were then used for clustering. Ward’s hierarchical clustering rendered the highest agglomerative coefficient (Ward = 0.81) for clustering of populations on phenotypic similarity compared with the other approaches [complete linkage = 0.71; average linkage (UPGMA) = 0.58; and single linkage = 0.49; Figure 6C]. The cutoff value at 8, indicated by horizontal line, resulted in three clusters. A PCA on quantitative trait data showed that the first three PCs explain 75.7% of the phenotypic variation among populations (PC1 = 43.1%, PC2 = 19.5%, and PC3 = 13.2%) supporting our grouping of chicken populations into three ecotypes (Supplementary Table 5).

A summary of cluster analyses (Table 2) shows that most of the populations of a specific ecotype are distributed within the same niche while only a few of them distributed elsewhere. Eight out of 12 populations from ecotype-I, three out of five populations from ecotype-II, and six out of nine populations from ecotype-III were correctly classified into their respective niches.

**TABLE 2:** Ecotype of Ethiopian indigenous chicken populations defined on phenotype and their respective niches as identified by species distribution models.

Ecotype	Populations	Distributed within the same niche	Distributed outside the niche
I	Fura, Dalecha, Birbirsa, Bengo, Almeshmesh, Badu, Didibe Kistana, Gazo, Meket, Weltane, Wahelo, Weledelelo	Fura, Dalecha, Birbirsa, Bengo, Almeshmesh, Badu, Didibe Kistana, Gazo	Meket, Weltane, Wahelo, Weldelelo
II	Lafinfedo, Hato, Melkajebdu, Arabo, Burkitu Obora	Lafinfedo, Hato, Melkajebdu,	Arabo, Burkitu Obora
III	Ebech, Shama, Parzeit, Gema, Zigh, Tumi, Rifenti Chabir, Sorobo, Gocha,	Shama, Parzeit, Zigh, Tumi, Rifenti Chabir, Sorobo	Ebech, Gema, Gocha

Matching between chicken ecotypes and different environmental classification methods was performed to establish a logical association between phenotypic distinctiveness and environmental selective pressures (Table 3). The environmental classification methods included SDMs, conventional (Dove, 1890), Official (MoA, 1998), and gradient-based agroecological classifications. The highest level of correct classification was performed by SDMs (64.5%), followed by environmental gradient (elevational cline) classification (57.3%). The higher correct classification level obtained by the SDM approach, suggests the potential influence of the selected environmental variables (*n* = 9) on shaping adaptive variation among Ethiopian indigenous chicken ecotypes.

**TABLE 3:** Comparison of methods to classify environments of Ethiopian indigenous chicken ecotypes (*n* = 3).

Classification method	Criteria for classification	No. of classes	No. of populations correctly classified (%)			Total no. of populations correctly classified (%)
			Ecotype-I	Ecotype-II	Ecotype-III	
SDM	Niche similarity	3	8 (66.7)	3 (60.0)	6 (66.7)	17 (64.5)
^*^Conventional AEs	Climatic classes (altitude, temperature, precipitation)	3	6 (66.7)	3 (33.3)	4 (50.0)	13 (50.0)
^§^ Official AEs	Temperature, soil type, plant growing period/moisture condition, land use	13	3 (33.3)	3 (33.3)	2 (25.0)	8 (30.7)
Environmental gradient	Elevational clines in distinct geographies	4	5 (55.5)	6 (66.6)	4 (50.0)	15 (57.3)

^*^Conventional agroecological classes (AEs) comprise three groups measured in m.a.s.l.: I = lowlands (400–1,800); II = 1,800–2,400; and III = 2,400–3,500 (Dove, 1890).

*^§^ Official AEs represent standard agroecologies of Ethiopia (MoA, 1998).*

### Environmental Variables Contribute to Phenotypic Differentiation

Having noticed that populations have differentiated distinctly in specific environments, we focused on predicting phenotypic values of ecotypes for the most discriminant quantitative traits within their respective niches under the influence of the selected environmental variables. Prediction of quantitative traits with GAMs in each of the three Ethiopian indigenous chicken ecotypes is presented in Table 4. Significant *p*-values were obtained for all the nine SDM identified environmental variables except for soil clay content. Five environmental variables (Bio18, Bio19, WVPM, and WVPA) had significant effect on differentiation of multiple traits. The traits selected by discriminant function for their usefulness in classification of populations into ecotypes had also the highest model fit (*R*-square adjusted values) explaining their importance in studying the influence of environmental variables on adaptive phenotypic variation.

**TABLE 4:** Prediction of quantitative traits with Generalized Additive Models (GAMs) in Ethiopian indigenous chicken ecotypes (*n* = 3).

Trait	^1^Fixed effects/linear term		Random effects/smoothing term									Model fit			
			Bio3	Bio4	Bio11	Bio18	Bio19	SRM	WVPM	WVPA	SCC	df	AIC	R-sq. (adj)	Deviance explained (%)
BL	Ecotype	Niches	*			*	***	*	***	***		14.9	1538.7	0.65	66.7
WS	Ecotype	Niches			***	***	*	*	***			13.6	1643.65	0.55	56.5
CL	Ecotype	Niches					*					8.0	2583.4	0.21	22.3
CW	Ecotype	Niches							***	***		9.3	2140.3	0.10	11.9
BW	Ecotype	Niches	*			**	*	***	***			12.5	-121.2	0.45	46.5
EW	Ecotype	Niches		***	.				***	***		16.3	1656.8	0.25	28.2
WW	Ecotype	Niches	*			**				**		10.0	2034.7	0.12	14.3
KL	Ecotype	Niches				*	**	.		**		11.9	1724.0	0.05	7.6

*Akaike information criterion (AIC) is a goodness of fit measure (likelihood or log-likelihood) that penalizes for complexity number of parameters or degree of freedoms). BL = body length; WS = wingspan; CL = comb length CW = comb width; BW = body weight; EW = earlobe width; WW = wattle width; and KL = beak length.*

*Bio3, Isothermality; Bio4, Temperature seasonality; Bio11, Mean temperature of coldest quarter; Bio18, Precipitation of warmest quarter; Bio19, Precipitation of coldest quarter; SRM, solar radiation of May; WVPM, water vapor pressure of May; WVPA, water vapor pressure of August; SCC, soil clay content.*

^1^Linear effect of ecotype is significant for all discriminating phenotypes. Significance codes: 0 “***” 0.001 “**” 0.01 “*” 0.05 “.”.

Ethiopian indigenous chicken ecotypes identified by SDMs showed significant quantitative trait variation (Table 5). Populations in ecotype-I had the smallest measurement for all traits while ecotype-II had the largest measurements for most traits. It is not possible to tell from the present results alone whether the performance exhibited by ecotypes is primarily attributable to their niche or their genetic background.

**TABLE 5:** Quantitative trait variation in Least Square Mean (Standard Error) among adult female Ethiopian indigenous chickens of different ecotypes defined by integrating SDMs with PDMs.

Ecotype^*^	LSMean (S.E.)									
	Hens (*n* = 380)								Cocks (*n* = 133)	
	BW	BL	WS	CL	CW	EW	WW	KL	BW	BL
I	1.01 (0.01)^b^	35.46 (0.22)^c^	38.78 (0.22)^b^	21.3 (0.62)^c^	7.95 (0.36)^b^	8.96 (0.2)^b^	16.89 (0.31)^c^	16.32 (0.20)	1.31 (0.05)^b^	38.89 (0.52)^b^
II	1.31 (0.02)^a^	39.13 (0.23)^a^	41.88 (0.24)^a^	30.21 (0.65)^a^	10.18 (0.37)^a^	10.9 (0.2)^a^	19.4 (0.32)^a^	16.46 (0.21)	1.78 (0.05)^a^	44.34 (0.54)^a^
III	1.28 (0.02)^a^	38.48 (0.24)^b^	42.03 (0.24)^a^	25.22 (0.66)^b^	8.82 (0.38)^b^	10.4 (0.20)^a^	18.48 (0.33)^b^	16.65 (0.22)	1.82 (0.05)^a^	44.49 (0.57)^a^

*BW = body weight (g); BL = body length(mm); WS = wingspan(mm); CL = comb length(mm); CW = comb width(mm); EW = earlobe width(mm); WW = wattle width(mm); and KL = beak length(mm).*

*^*a,b,c*^Means with different superscripts within the same column are significantly (*p* < 0.05) different.*

^*^Ecotypes were highly significant from each other (*p* < 0.0001) for all phenotypic measurements except for KL in hens (*p* = 0.5393).

**FIGURE 7:**
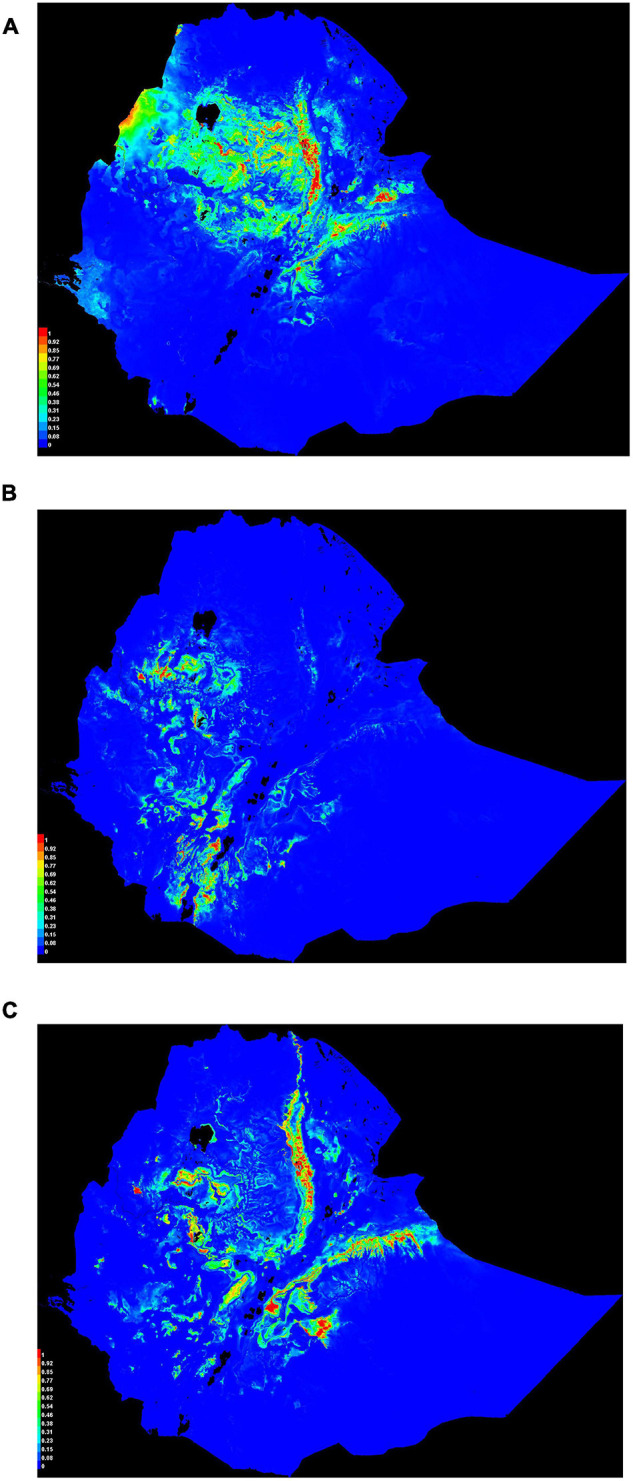
Suitability maps of three Ethiopian chicken ecotypes. Colors toward red spectrum indicate more suitable conditions. **(A)** Ecotype-I. **(B)** Ecotype-II. **(C)** Ecotype-III.

Habitat suitability maps for Ethiopian indigenous chicken ecotypes (Figure 7) illustrate ideal environmental conditions that vary spatially between ecotypes. Chickens of ecotype-I (Figure 7A) are mainly distributed in central and northwest Ethiopia, ecotype-II (Figure 7B) are distributed in the west and southwest, while ecotype-III (Figure 7C) are distributed in eastern and northeastern Ethiopia. Areas of the country characterized by adverse environmental conditions due to their extreme temperature, high solar radiation, and low precipitation are shown as least suitable. This result conforms to the available census data which shows regions in the country with more friendly climate to chickens are more populated by the species (CSA, 2017).

**FIGURE 8:**
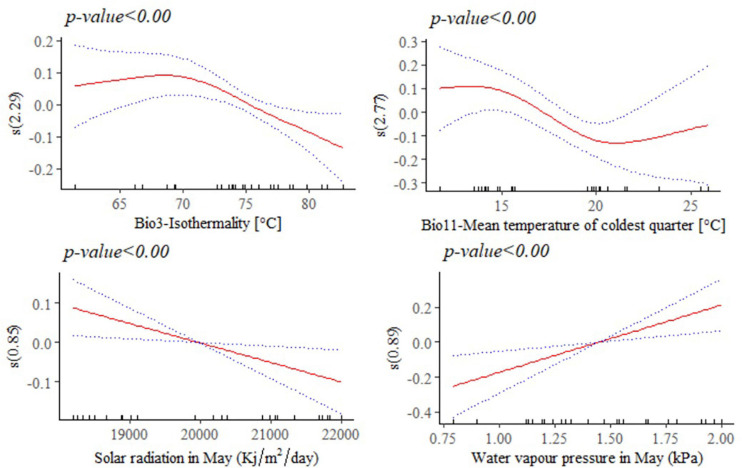
Generalized additive model partial dependence plots for live body weight (kg) in female indigenous chickens. Each plot shows a covariate and the partial dependence of adult live body weight in the context of the model. The *y* axis shows the mean of observed change in live body weight and the *x* axis the covariate interval. The blue line represents the 95% confidence interval; Red line, mean of observed change in live body weight; s, smoothed variable; and *( )*, effective degrees of freedom.

**FIGURE 9:**
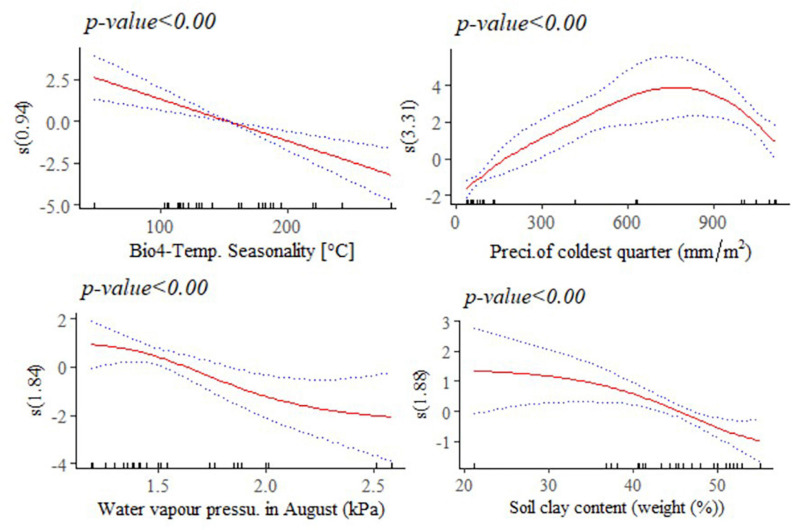
Generalized additive model partial dependence plots for body length (mm) in female indigenous chickens. Each plot shows a covariate and the partial dependence of adult live body weight in the context of the model. The *y* axis shows the mean of observed change in live body length and the *x* axis the covariate interval. The blue line represents the 95% confidence interval; Red line, mean of observed change in live body weight; s, smoothed variable; and *( )*, effective degrees of freedom.

The response of adult live body weight (BW) and BL in female indigenous chickens to some of the significant environmental variables (*p* < 0.001) are presented in Figures 8, 9. The relationship between BW and solar radiation, and BW and water vapor pressure in May (kPa) is linear while its relationship with isothermality is non-linear (Figure 8). Isothermality quantifies how large the day-to-night temperatures oscillate relative to the annual oscillations. An isothermal value of 100 indicates the diurnal temperature range is equivalent to the annual temperature range, while anything less than 100 indicates a smaller level of temperature variability within an average month relative to the year (O’Donnell and Ignizio, 2012). Our results suggest that BW is less influenced by smaller temperature fluctuations within a month relative to the year. On the other hand, solar radiation above 18,000 kJ/m^2^/day is stressful and has negative and linear effect on female BW. The relationship between bodyweight and mean temperature of the coldest quarter is more complex, showing that the mean temperatures during the coldest 3 months of the year is less useful to examine how this variable affects adult live BW.

A non-linear relationship is noted between BL and water vapor pressure in August (kPa), and between BL and precipitation of the coldest quarter (mm/m^2^). Temperature seasonality had a negative and linear relationship with this trait. Temperature seasonality is a measure of temperature change over the course of the year. Our result indicates that higher standard deviation in the mean monthly temperature is associated with smaller BL, a trait which is strongly correlated with live BW. Precipitation of the coldest quarter is a quarterly index which approximates the total precipitation that prevails during the 3 months of the year. Accelerated mean change in BL, in the context of the model was seen up to 700 mm/m^2^ of precipitation in the coldest quarter. Precipitation above this threshold might be related with less availability of scavenging feed resources and more prevalence of diseases and parasites, having adverse effects on this trait. Biologically speaking, water vapor pressure is a function of temperature and pressure. Negative relation is noted between this environmental variable and BL, probably because of the stressful situation (e.g., lower feed intake) it creates on the animals. A non-linear reduction was observed in BL for higher soil clay content above 20% which may have a relationship with the type of vegetation and land use pattern in those areas (Figure 9).

## Discussion

Sustainable livestock production particularly in the tropics requires adaptive genotypes which can withstand the undesirable effects of climate change and produce optimally (Fleming et al., 2017; Bettridge et al., 2018). Ecological variables vary in terms of their influences on organisms as inducers of local adaptation. Knowledge of ecological factors responsible for adaptive variation should be the first step to design selective breeding programs on indigenous livestock, plan crossbreeding with improved genotypes, or introduce new genotypes from a different environment (Fleming et al., 2017; Bettridge et al., 2018; Birhanu et al., 2021; Gheyas et al., 2021).

We have applied distribution models to identify the most important environmental factors associated with habitat suitability and phenotypic differentiation in indigenous populations of chickens. Previous studies indicated that populations differentiate phenotypically and genetically in response to the environment (Schmid and Guillaume, 2017; Smith et al., 2017). A tight relation is expected between environmental elements (e.g., precipitation, temperature, radiation, and elevation) and livestock population dynamics (Alemayehu and Getu, 2016; Getachew et al., 2016) in Ethiopia.

Precipitation of the warmest and the coldest quarters, soil clay content, temperature seasonality, solar radiation, water vapor pressure, and mean temperature of the coldest quarter, were identified by SDMs as the most important variables associated with habitat suitability in Ethiopian indigenous chickens. Precipitation is associated with types and amounts of crops cultivated; availability of scavenging feed resources and edible soil fauna; disease prevalence, and predation. Precipitation and temperature were also identified as most important contributors to local adaptation in African chickens (Fleming et al., 2017; Bettridge et al., 2018; Gheyas et al., 2021). The BW of Horro, Koekoek, Sasso, and SRIR chickens distributed to different regions of Ethiopia was best predicted by variables associated with temperature and precipitation (Lozano-Jaramillo A. et al., 2019). Clay content is a proxy for soil fertility and has impacts on feed availability for poultry. Through their physical and chemical properties, clay minerals can be expected to have more nutrient reserves in the tropics (Landon, 2014; Kome et al., 2019).

All the nine environmental variables selected for their association with habitat suitability by SDMs had significant effects on differentiation of quantitative traits. The influence of isothermality (Bio3), temperature (Bio4 and Bio11), precipitation (Bio19), solar radiation, and water vapor pressure on trait differentiation may be related with adaptive physiology of chickens, in terms of their biological response to extremes in relative humidity and heat stress. Lozano-Jaramillo A. et al. (2019) and Alemu et al. (2021) have also observed effects of precipitation and temperature on improved chicken breeds introduced to smallholder farmers in Ethiopia.

We classified the Ethiopian indigenous chicken sample populations into three ecotypes and compared their respective performances. Homogenous clusters for measured quantitative traits and their overlaps with distinct niches were used to define ecotypes. Unlike previous efforts made to group Ethiopian indigenous chicken populations on qualitative phenotypes such as comb shape, and feather color (Melesse and Negesse, 2011; FAO, 2012; Negassa et al., 2014; Getachew et al., 2016; Overdijk, 2019), the definition of ecotypes in the present study integrated phenotypic and environmental information. This process included identification of the most contributing environmental variables for habitat suitability, grouping of sample locations into specific niches based on their environmental similarity, and selection of the most useful quantitative traits for population classification purposes.

Phenotypic distribution models, in a form of non-linear GAMs were demonstrated as an innovative approach to integrate environmental and phenotypic information and study their relationships. GAMs relax the assumptions of linear models such as GLMs and achieve acceptable goodness of fit. Such a non-linear data structure would have been missed otherwise (Wiley and Wiley, 2019). PDMs were used to complement predictions of SDMs in studying responses of prairie grass to climate change (Smith et al., 2017).

The use of SDMs is unchartered territory for livestock scientists. Limitations are expected in their use on domesticated species because of human interference influencing the natural distribution of the study populations. While existing SDMs alone do not seem appropriate to study breeds recently introduced into a new environment artificially, the models are applicable for those studying local adaptation among indigenous populations of livestock which have lived in their environment for hundreds of generations or more and have experienced significant selective pressures. Predictive ability of machine learning algorithms on domesticated species can be improved if they incorporate more data in addition to presence–absence information and harness sophisticated algorithms. Boosted regression trees and random forests as well as generalized additive and linear mixed models have improved prediction of SDMs in other species (Shirk et al., 2018).

Several evolutionary processes shape genetic and phenotypic differentiation, including the joint effects of environment (phenotypic plasticity), gene flow, and natural selection (Schmid and Guillaume, 2017). It is not clear from the present study whether the phenotypic differentiation that ensued between indigenous chicken ecotypes is the result of differentiation in allele frequencies. An integrated framework including environmental, phenotypic, and genomic analysis is needed to unravel the genetic basis of phenotypic differentiation among populations and ecotypes of these chickens. If the phenotype is directly influenced by the environment, genetic, and phenotypic differentiations can be decoupled (Crispo, 2008; Schmid and Guillaume, 2017). Improvements in predictive ability of models is also achieved when SDMs are used along with phenotypic and genomic information in landscape genetics and genomics studies (Joost et al., 2007; Gotelli and Stanton-Geddes, 2015; Razgour, 2015).

The present study demonstrated how SDM-identified environmental information can be integrated with PDMs to define ecotypes, predict quantitative traits, and understand the ecological roots of phenotypic differentiation. Considering the environmental influences of economically important quantitative traits, such as live BW, improves the estimation of breeding values and assists in the development of improved breeds suited to smallholder farmers. Differences in performance among ecotypes in the different niches will also mean evaluations of performance and yield stability across environments are pertinent in breeding and development programs designed for low- and medium-input poultry production systems of the tropics. Prospects of further use for SDMs and PDMs in livestock include definition of agroecologies, estimation of genotype by agroecology interactions, multi-environment performance evaluations, and prediction of performance under present and future production scenarios (e.g., climate change).

## Data Availability Statement

The raw data supporting the conclusions of this article will be made available by the authors, without undue reservation.

## Ethics Statement

The animal study was reviewed and approved by Institutional Animal Care and Use Committee (IACUC), International Livestock Research Institute (ILRI).

## Author Contributions

FGK, JB, HK, OH, and TD conceived the ideas and designed the study. FGK selected sample populations, collected metadata, performed the phenotyping of chickens and data analysis, and wrote the manuscript. JB and SA supported in the GAMs analysis. HK and JB provided useful comments and suggestions and helped improve the data analysis and draft the manuscript. TD, HK, OH, and JB secured funding. All authors critically revised the manuscript and gave final approval for publication.

## Conflict of Interest

The authors declare that the research was conducted in the absence of any commercial or financial relationships that could be construed as a potential conflict of interest.

## Publisher’s Note

All claims expressed in this article are solely those of the authors and do not necessarily represent those of their affiliated organizations, or those of the publisher, the editors and the reviewers. Any product that may be evaluated in this article, or claim that may be made by its manufacturer, is not guaranteed or endorsed by the publisher.

## Footnotes

1 http://www.worldclim.org/; version 2

2
http://www.diva-gis.org/gdata

## Supplementary Material

Supplementary Table 1 Geographic distribution of 26 Ethiopian indigenous chicken sample populations. ^∗^Traditional agroecological classes (AEs) comprise three groups measured in m.a.s.l.: I = lowlands (400–1,800); II = 1,800–2,400; and III = 2,400–3,500 (Dove, 1890). ^§^ Official AEs represent standard agroecologies of Ethiopia (MoA, 1998).

Supplementary Table 2 Environmental (climatic, soil, vegetation type) variables obtained for the locations of indigenous Ethiopian chicken sample populations.

Supplementary Table 3 ENMeval table results for all combinations of features and betamultipliers. ^∗^Feature classes: L, linear; Q, quadratic; H, hinge; P, product; and T, threshold. ^§^ RM, Regularization multiplier.

Supplementary Table 4 Quantitative traits measured on individual hens and cocks (*n* = 513) in indigenous Ethiopian chicken sample populations.

Supplementary Table 5 Principal Component Analysis (PCA) results for female quantitative traits.
