# Supplementary material for: Species and Phenotypic Distribution Models Reveal Population Differentiation in Ethiopian Indigenous Chickens
Source: Front Genet. 2021 Sep 8;12:723360. doi: 10.3389/fgene.2021.723360 (PMC8456010; doi:10.3389/fgene.2021.723360)
Supplement: Supplementary Table 1 — Geographic distribution of 26 Ethiopian indigenous chicken sample populations. ∗Traditional agroecological classes (AEs) comprise three groups measured in m.a.s.l.: I = lowlands (400–1,800); II = 1,800–2,400; and III = 2,400–3,500 (Dove, 1890). § Official AEs represent standard agroecologies of Ethiopia (MoA, 1998). [file Table_1.docx]

**Supplementary Table 1**. Geographic distribution of 26 Ethiopian indigenous chicken sample populations

| **Population (code)** | **District** | **Spatial gradient** | ***Traditional AEZ** | **^§^Official AEZ** | **Regional State** | **Geolocation** | | **Elevation (m.a.s.l.)** |
| --- | --- | --- | --- | --- | --- | --- | --- | --- |
|  |  |  |  |  |  | **Long (E)** | **Lat (N)** |  |
| Gazo (1) | Gazo | I | III | M3 | Amhara | 39.12 | 11.68 | 3175 |
| Meket (2) | Meket | I | III | M3 | Amhara | 38.75 | 11.72 | 2895 |
| Wahelo (3) | Tehuledere | I | II | SM2 | Amhara | 39.65 | 11.29 | 2150 |
| Weldelelo (4) | Tehuledere | I | II | SM2 | Amhara | 39.71 | 11.26 | 2060 |
| Arabo (7) | Kalu | I | I | M2 | Amhara | 39.93 | 11.16 | 1525 |
| Fura (6) | Bati | I | I | SM1 | Amhara | 40.06 | 11.18 | 1400 |
| Hato (5) | Bati | I | I | SM1 | Amhara | 40.05 | 11.12 | 1205 |
| Badu (11) | Girawa | II | III | H3 | Oromia | 41.86 | 9.87 | 2464.5 |
| Birbirsa (10) | Girawa | II | III | H3 | Oromia | 41.76 | 9.21 | 2437.8 |
| Lafinfedo (12) | Jarso | II | II | SM2 | Oromia | 42.23 | 9.44 | 2175.8 |
| Melkajebdu (13) | Jarso | II | II | SM2 | Oromia | 42.28 | 9.53 | 2017.4 |
| Dalecha (8) | Meiso | II | I | SA1 | Oromia | 40.65 | 9.21 | 1522.6 |
| Weltane (9) | Meiso | II | I | SA1 | Oromia | 40.87 | 9.26 | 1295.6 |
| Tumi (15) | Wombera | III | III | SM3 | Benishangul-Gumuz | 35.71 | 10.59 | 2527.8 |
| Ebech (14) | Wombera | III | II | SH2 | Benishangul-Gumuz | 35.71 | 10.64 | 2017.4 |
| Parzeit (17) | Dibate | III | I | SH1 | Benishangul-Gumuz | 36.23 | 10.67 | 1581.2 |
| Zigh (16) | Dibate | III | I | M1 | Benishangul-Gumuz | 36.16 | 10.68 | 1421.4 |
| Almeshmesh (19) | Guba | III | I | SM1 | Benishangul-Gumuz | 35.3 | 11.8 | 662.7 |
| Bengo (18) | Guba | III | I | SM1 | Benishangul-Gumuz | 35.08 | 11.64 | 407.4 |
| Didibe Kistana (20) | Horro | IV | III | SH3 | Oromia | 37.17 | 9.59 | 2467.7 |
| Burkitu Obora (21) | Horro | IV | III | SH3 | Oromia | 37.04 | 9.5 | 2421 |
| Rifenti Chabir (22) | Horro Buluk | IV | III | SH3 | Oromia | 37.07 | 9.61 | 2710 |
| Gocha (23) | Karat zuria | IV | I | H1 | SNNPR | 37.39 | 5.34 | 1583.3 |
| Sorobo (24) | Karat zuria | IV | I | H1 | SNNPR | 37.44 | 5.42 | 1241.8 |
| Shama (25) | Chencha | IV | II | H2 | SNNPR | 37.51 | 6.17 | 2185.7 |
| Gema (26) | Chencha | IV | III | SH3 | SNNPR | 37.65 | 6.29 | 2445.4 |

*****Traditional agroecological classes (AEs) comprise three groups measured in m.a.s.l.: I=lowlands (400-1800); II=1800-2400; III=2400-3500 (Dove, 1890). **^§^** Official AEs represent standard agroecologies of Ethiopia (MoA, 1998).
