# Supplementary material for: Species and Phenotypic Distribution Models Reveal Population Differentiation in Ethiopian Indigenous Chickens
Source: Front Genet. 2021 Sep 8;12:723360. doi: 10.3389/fgene.2021.723360 (PMC8456010; doi:10.3389/fgene.2021.723360)
Supplement: Supplementary Table 2 — Environmental (climatic, soil, vegetation type) variables obtained for the locations of indigenous Ethiopian chicken sample populations. [file Table_2.docx]

**Supplementary Table 2.** Environmental (climatic, soil, vegetation type) variables obtained for the locations of indigenous Ethiopian chicken sample populations

| Type | Variable | Units | Link |
| --- | --- | --- | --- |
| Climatic | Bio1-Annual mean temperature | ^0^C | <http://www.worldclim.org/version2> |
|  | Bio2-Mean diurnal range of temperature | ^0^C |  |
|  | Bio3-Isothermality (Bio2/Bio7) (x100) | ^0^C |  |
|  | Bio4-Temperature seasonality (SD x100) | ^0^C |  |
|  | Bio5-Max temperature of warmest month | ^0^C |  |
|  | Bio6-Min temperature of coldest month | ^0^C |  |
|  | Bio7-Temperature annual range (Bio5-Bio6) | ^0^C |  |
|  | Bio8-Mean temperature of wettest quarter | ^0^C |  |
|  | Bio9-Mean temperature of driest quarter | ^0^C |  |
|  | Bio10-Mean temperature of warmest quarter | ^0^C |  |
|  | Bio11-Mean temperature of coldest quarter | ^0^C |  |
|  | Bio12-Annual precipitation | mm/m^2^ |  |
|  | Bio13-Precipitation of wettest month | mm/m^2^ |  |
|  | Bio14-Precipitation of driest month | mm/m^2^ |  |
|  | Bio15-Precipitation seasonality (CV) | mm/m^2^ |  |
|  | Bio16-Precipitation of wettest quarter | mm/m^2^ |  |
|  | Bio17-Precipitation of driest quarter | mm/m^2^ |  |
|  | Bio18-Precipitation of warmest quarter | mm/m^2^ |  |
|  | Bio19-Precipitation of coldest quarter | mm/m^2^ |  |
|  | Solar radiation of the month of May | Kj m^-2^ day^-1^ |  |
|  | Solar radiation of the month of August | Kj m^-2^ day^-1^ |  |
|  | Water vapor pressure for the month of May | kPa |  |
|  | Water vapor pressure for the month of August | kPa |  |
|  | Bioelevation | m.a.s.l. | <http://www.diva-gis.org/datadown> |
| Soil | Derived available soil water capacity | v% | [www.soilgrids.org](http://www.soilgrids.org/) |
|  | Soil pH (at 30cm depth) | pH (x10 in H_2_O) | <https://www.isric.org/projects/soil-property-maps-africa-250-m-resolution> |
|  | Soil clay content | weight (%) |  |
|  | Soil silt content | weight (%) |  |
|  | Soil sand content | weight (%) |  |
|  | Soil cation exchange capacity | cmol+kg^−1^ |  |
|  | Soil organic carbon content | % (g kg^−1^) |  |
|  | Bulk density (<2mm) | kg dm^−3^ |  |
| Vegetation | Crop land extent | area (ha) | <https://croplands.org/downloadLPDAAC> |
|  | Land cover | % | <https://www.usgs.gov/media/files/africa-land-cover-characteristics-data-base-version-20> |
