# Supplementary material for: Species and Phenotypic Distribution Models Reveal Population Differentiation in Ethiopian Indigenous Chickens
Source: Front Genet. 2021 Sep 8;12:723360. doi: 10.3389/fgene.2021.723360 (PMC8456010; doi:10.3389/fgene.2021.723360)
Supplement: Supplementary Table 3 — ENMeval table results for all combinations of features and betamultipliers. ∗Feature classes: L, linear; Q, quadratic; H, hinge; P, product; and T, threshold. § RM, Regularization multiplier. [file Table_3.docx]

**Supplementary Table 3.** ENMeval table results for all combinations of features and betamultipliers

|  | Features^*^ | RM^§^ | train.AUC | avg.test.AUC | var.test.AUC | var.diff.AUC | avg.test.orMTP | var.test.or  10pct | AICc | delta.  AICc |
| --- | --- | --- | --- | --- | --- | --- | --- | --- | --- | --- |
| 1 | H | 0.5 | 0.986 | 0.935 | 0.159 | 0.001 | 0.004 | 0.006 | 6587.082 | 470.475 |
| 2 | LQH | 0.5 | 0.983 | 0.980 | 0.001 | 0.001 | 0.004 | 0.002 | 6717.449 | 600.843 |
| 3 | HQP | 0.5 | 0.986 | 0.937 | 0.145 | 0.001 | 0.004 | 0.002 | 6539.692 | 423.085 |
| 4 | HQC | 0.5 | 0.987 | 0.937 | 0.067 | 0.001 | 0.004 | 0.003 | 6520.487 | 403.881 |
| 5 | LQHP | 0.5 | 0.984 | 0.937 | 0.145 | 0.001 | 0.004 | 0.004 | 6559.791 | 443.184 |
| 6 | LQHPT | 0.5 | 0.984 | 0.937 | 0.145 | 0.001 | 0.004 | 0.004 | 6559.791 | 443.184 |
| 7 | H | 1 | 0.985 | 0.935 | 0.158 | 0.001 | 0.004 | 0.003 | 6769.529 | 652.922 |
| 8 | LQH | 1 | 0.986 | 0.936 | 0.160 | 0.001 | 0.008 | 0.003 | 6688.029 | 571.423 |
| 9 | HQP | 1 | 0.985 | 0.980 | 0.001 | 0.001 | 0.008 | 0.002 | 6706.115 | 589.509 |
| 10 | HQC | 1 | 0.985 | 0.935 | 0.159 | 0.001 | 0.004 | 0.004 | 6724.506 | 607.899 |
| 11 | LQHP | 1 | 0.987 | 0.979 | 0.002 | 0.001 | 0.004 | 0.004 | 6738.374 | 621.768 |
| 12 | LQHPT | 1 | 0.987 | 0.979 | 0.002 | 0.001 | 0.004 | 0.004 | 6738.374 | 621.768 |
| 13 | H | 1.5 | 0.985 | 0.981 | 0.000 | 0.000 | 0.004 | 0.002 | 6795.165 | 678.559 |
| 14 | LQH | 1.5 | 0.984 | 0.980 | 0.000 | 0.000 | 0.004 | 0.002 | 6774.314 | 657.708 |
| 15 | HQP | 1.5 | 0.985 | 0.982 | 0.000 | 0.000 | 0.004 | 0.002 | 6643.753 | 527.146 |
| 16 | HQC | 1.5 | 0.985 | 0.980 | 0.000 | 0.000 | 0.004 | 0.002 | 6813.500 | 696.893 |
| 17 | LQHP | 1.5 | 0.986 | 0.982 | 0.000 | 0.000 | 0.004 | 0.002 | 6752.763 | 636.156 |
| 18 | LQHPT | 1.5 | 0.986 | 0.982 | 0.000 | 0.000 | 0.004 | 0.002 | 6752.763 | 636.156 |
| 19 | H | 2 | 0.982 | 0.977 | 0.000 | 0.000 | 0.004 | 0.003 | 6588.341 | 471.734 |
| 20 | LQH | 2 | 0.981 | 0.977 | 0.000 | 0.000 | 0.004 | 0.004 | 6366.471 | 249.864 |
| 21 | HQP | 2 | 0.983 | 0.979 | 0.000 | 0.000 | 0.004 | 0.003 | 6378.248 | 261.641 |
| 22 | HQC | 2 | 0.981 | 0.977 | 0.000 | 0.000 | 0.004 | 0.004 | 6384.714 | 268.108 |
| 23 | LQHP | 2 | 0.984 | 0.979 | 0.000 | 0.000 | 0.004 | 0.002 | 6406.483 | 289.877 |
| 24 | LQHPT | 2 | 0.984 | 0.979 | 0.000 | 0.000 | 0.004 | 0.002 | 6406.483 | 289.877 |
| 25 | H | 2.5 | 0.977 | 0.972 | 0.000 | 0.000 | 0.004 | 0.006 | 6326.606 | 209.999 |
| 26 | LQH | 2.5 | 0.978 | 0.973 | 0.000 | 0.000 | 0.004 | 0.003 | 6299.631 | 183.025 |
| 27 | HQP | 2.5 | 0.981 | 0.976 | 0.000 | 0.000 | 0.004 | 0.003 | 6339.137 | 222.530 |
| 28 | HQC | 2.5 | 0.978 | 0.973 | 0.000 | 0.000 | 0.000 | 0.005 | 6277.624 | 161.018 |
| 29 | LQHP | 2.5 | 0.980 | 0.975 | 0.000 | 0.000 | 0.004 | 0.002 | 6324.046 | 207.440 |
| 30 | LQHPT | 2.5 | 0.980 | 0.975 | 0.000 | 0.000 | 0.004 | 0.002 | 6324.046 | 207.440 |
| 31 | H | 3 | 0.973 | 0.967 | 0.001 | 0.000 | 0.000 | 0.006 | 6223.526 | 106.920 |
| 32 | LQH | 3 | 0.974 | 0.967 | 0.000 | 0.000 | 0.004 | 0.004 | 6138.641 | 22.034 |
| 33 | HQP | 3 | 0.972 | 0.965 | 0.001 | 0.000 | 0.004 | 0.004 | 6116.607 | 0.000 |
| 34 | LQHPT | 3 | 0.977 | 0.970 | 0.000 | 0.000 | 0.004 | 0.005 | 6182.527 | 65.920 |
| 35 | HQC | 3 | 0.973 | 0.967 | 0.000 | 0.000 | 0.004 | 0.006 | 6157.147 | 40.540 |
| 36 | LQHP | 3 | 0.977 | 0.970 | 0.000 | 0.000 | 0.004 | 0.005 | 6182.527 | 65.920 |
| 37 | H | 3.5 | 0.969 | 0.961 | 0.001 | 0.001 | 0.000 | 0.005 | 6266.127 | 149.520 |
| 38 | LQH | 3.5 | 0.968 | 0.960 | 0.001 | 0.000 | 0.004 | 0.005 | 6137.779 | 21.172 |
| 39 | HQP | 3.5 | 0.972 | 0.965 | 0.001 | 0.000 | 0.004 | 0.003 | 6131.879 | 15.273 |
| 40 | HQC | 3.5 | 0.969 | 0.961 | 0.001 | 0.000 | 0.004 | 0.007 | 6176.550 | 59.944 |
| 41 | LQHP | 3.5 | 0.977 | 0.971 | 0.000 | 0.000 | 0.000 | 0.003 | 6126.377 | 9.771 |
| 42 | LQHPT | 3.5 | 0.972 | 0.965 | 0.001 | 0.000 | 0.004 | 0.004 | 6116.607 | 0.000 |
| 43 | H | 4 | 0.964 | 0.954 | 0.002 | 0.001 | 0.004 | 0.006 | 6245.073 | 128.466 |
| 44 | LQH | 4 | 0.962 | 0.952 | 0.001 | 0.001 | 0.004 | 0.002 | 6161.555 | 44.948 |
| 45 | HQP | 4 | 0.968 | 0.959 | 0.001 | 0.000 | 0.004 | 0.004 | 6134.046 | 17.440 |
| 46 | HQC | 4 | 0.962 | 0.953 | 0.001 | 0.001 | 0.008 | 0.006 | 6202.096 | 85.490 |
| 47 | LQHP | 4 | 0.967 | 0.959 | 0.001 | 0.000 | 0.004 | 0.005 | 6162.405 | 45.799 |
| 48 | LQHPT | 4 | 0.967 | 0.959 | 0.001 | 0.000 | 0.004 | 0.005 | 6162.405 | 45.799 |

^*^Feature classes: L = linear, Q = quadratic, H = hinge, P = product and T = threshold. ^§^RM: Regularization multiplier
