# Supplementary material for: Species and Phenotypic Distribution Models Reveal Population Differentiation in Ethiopian Indigenous Chickens
Source: Front Genet. 2021 Sep 8;12:723360. doi: 10.3389/fgene.2021.723360 (PMC8456010; doi:10.3389/fgene.2021.723360)
Supplement: Supplementary Table 4 — Quantitative traits measured on individual hens and cocks (n = 513) in indigenous Ethiopian chicken sample populations. [file Table_4.docx]

**Supplementary Table 4**. Quantitative traits measured on individual hens and cocks (*n*=513) in indigenous Ethiopian chicken sample populations

| **No.** | **Trait** | **Details** | **Unit** | **Equipment** | **Accuracy** | **Adaptive roles** |
| --- | --- | --- | --- | --- | --- | --- |
| 1. | Adult live body weight (BW) | fasting weight | g | digital balance | 10g | proxy for body size; thermoregulation  (Fayeye et al., 2014) (Washburn et al., 1992) |
| 2. | Wingspan (WS) | on top between the longest primaries with wings stretched | mm | measuring tape | 1mm | thermoregulation (Gerken et al., 2006) |
| 3. | Body length (BL) | from tip of the beak to the tail (without feathers) | mm | measuring tape | 1mm | proxy for body size; thermoregulation  (Semakula et al., 2011) |
| 4. | Chest circumference (CC) | at the hind breast | mm | measuring tape | 1mm | proxy for body size; thermoregulation  (Semakula et al., 2011) |
| 5. | Shank length (SL) | length of the tarsometatarsus | mm | ImageJ | 0.1mm | proxy for body size; diet and foraging behaviour; thermoregulation (Semakula et al., 2011) |
| 6 | Shank circumference (SC) | at the middle of the tarsometatarsus | mm | measuring tape | 1mm |  |
| 7 | Beak width (KW) | from the insertion of the beak in the skull and perpendicular until the end of the inferior mandible | mm | ImageJ | 0.1mm | Diet and foraging behaviour; thermoregulation  (Friedman et al., 2017); (Norazlimi and Ramli, 2015); (Ryeland et al., 2017) |
| 8 | Beak length (KL) | tip of the beak to insertion of the beak into the skull | mm | ImageJ | 0.1mm |  |
| 9 | Beak area (KA) | from the insertion of the beak in the skull and perpendicular until the end of the inferior mandible | mm | ImageJ | 0.1mm |  |
| 10 | Comb length (CL) | between the insertion of the comb in the beak and the end of the comb’s lobe | mm | ImageJ | 0.1mm | Thermoregulation (Sturkie, 1965) (Gerken et al., 2006) (Wright et al., 2009) |
| 11 | Comb width (CW) | from the tip of the central spike until insertion of the comb in the skull | mm | ImageJ | 0.1mm |  |
| 12 | Comb area (CA) | area of two-dimensional comb | mm^2^ | ImageJ | 0.1mm^2^ |  |
| 13 | Wattle length (WL) | from insertion of the right wattle into the beak to the end of the wattle | mm | ImageJ | 0.1mm | thermoregulation (Sturkie, 1965) (Gerken et al., 2006; Wright et al., 2009) (Hester et al., 2015) |
| 14 | Wattle width (WW) | the second maximum dimension of the wattle perpendicular to the length | mm | ImageJ | 0.1mm |  |
| 15 | Wattle area (WA) | area of two-dimensional wattle | mm^2^ | ImageJ | 0.1mm^2^ |  |
| 16 | Earlobe length (EL) | maximum length, keeping the head of the chicken perpendicular to the neck | mm | ImageJ | 0.1mm | Thermoregulation (Luo et al., 2018) |
| 17 | Earlobe width (EW) | the second maximum dimension | mm | ImageJ | 0.1mm |  |
| 18 | Earlobe area (EA) | two-dimensional area | mm^2^ | ImageJ | 0.1mm^2^ |  |
| 19 | Ocular area (OA) | two-dimensional | mm^2^ | ImageJ | 0.1mm^2^ | feeding habit; thermoregulation (Gerken et al., 2006) |
