# Supplementary material for: Species and Phenotypic Distribution Models Reveal Population Differentiation in Ethiopian Indigenous Chickens
Source: Front Genet. 2021 Sep 8;12:723360. doi: 10.3389/fgene.2021.723360 (PMC8456010; doi:10.3389/fgene.2021.723360)
Supplement: Supplementary Table 5 — Principal Component Analysis (PCA) results for female quantitative traits. [file Table_5.docx]

**Supplementary Table 5**. Principal Component Analysis (PCA) results for female quantitative traits

|  | **Eigen value** | **Variance percent** | **Cumulative variance percent** |
| --- | --- | --- | --- |
| Dim.1 | 3.4 | 43.1 | 43.1 |
| Dim.2 | 1.6 | 19.5 | 62.6 |
| Dim.3 | 1.1 | 13.2 | 75.7 |
| Dim.4 | 0.7 | 8.6 | 84.3 |
| Dim.5 | 0.6 | 7.3 | 91.7 |
| Dim.6 | 0.3 | 4.3 | 95.9 |
| Dim.7 | 0.2 | 2.2 | 98.1 |
| Dim.8 | 0.2 | 1.9 | 100 |
