# Supplementary figures and images for: Species and Phenotypic Distribution Models Reveal Population Differentiation in Ethiopian Indigenous Chickens
Source: Front Genet. 2021 Sep 8;12:723360. doi: 10.3389/fgene.2021.723360 (PMC8456010; doi:10.3389/fgene.2021.723360)

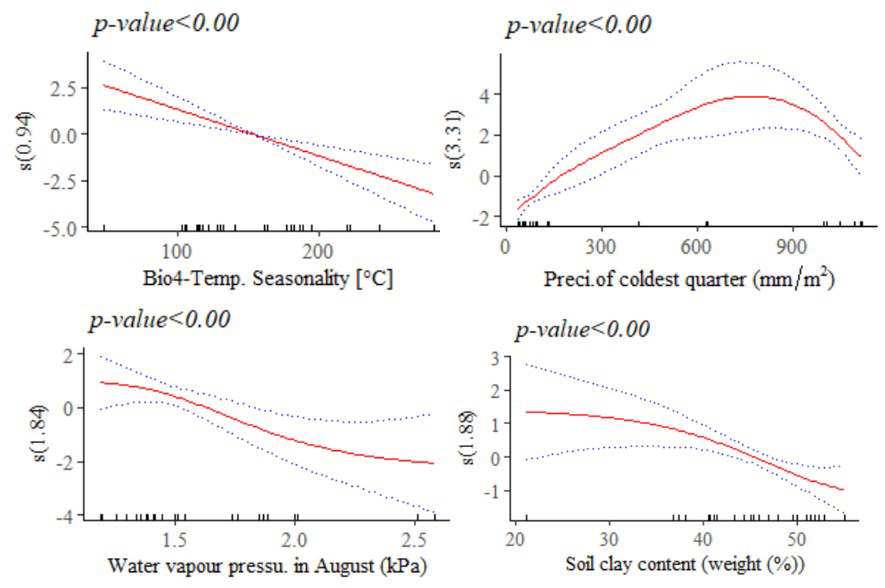

Supplement: Supplementary file 6 [file Image_1.JPEG]

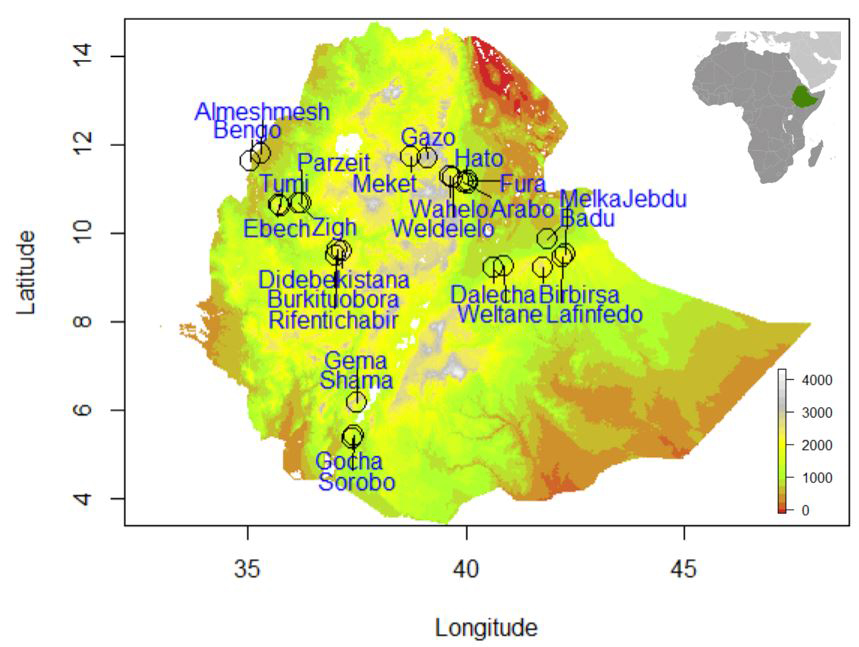

Supplement: Supplementary file 7 [file Image_2.JPEG]

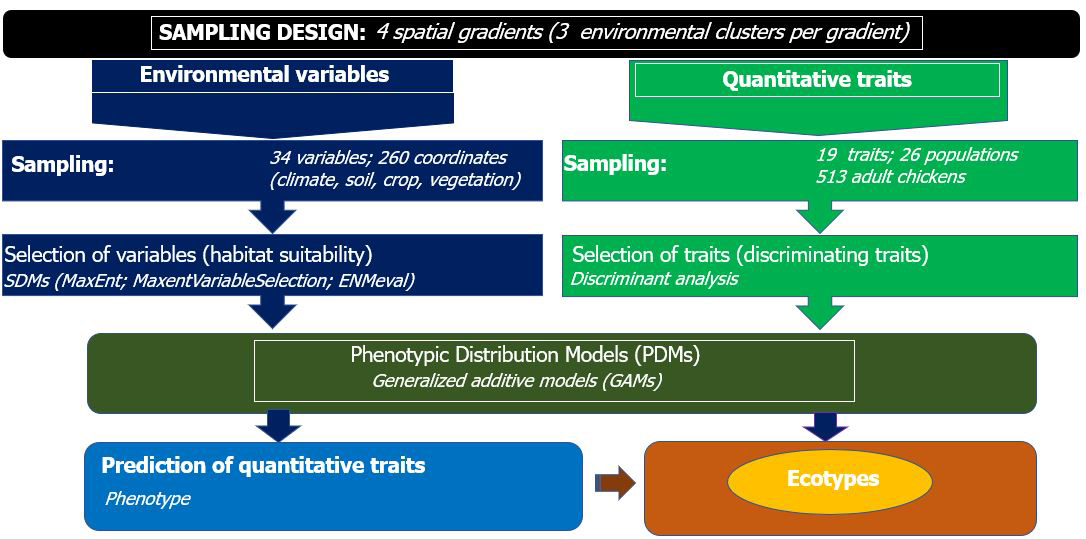

Supplement: Supplementary file 8 [file Image_3.JPEG]

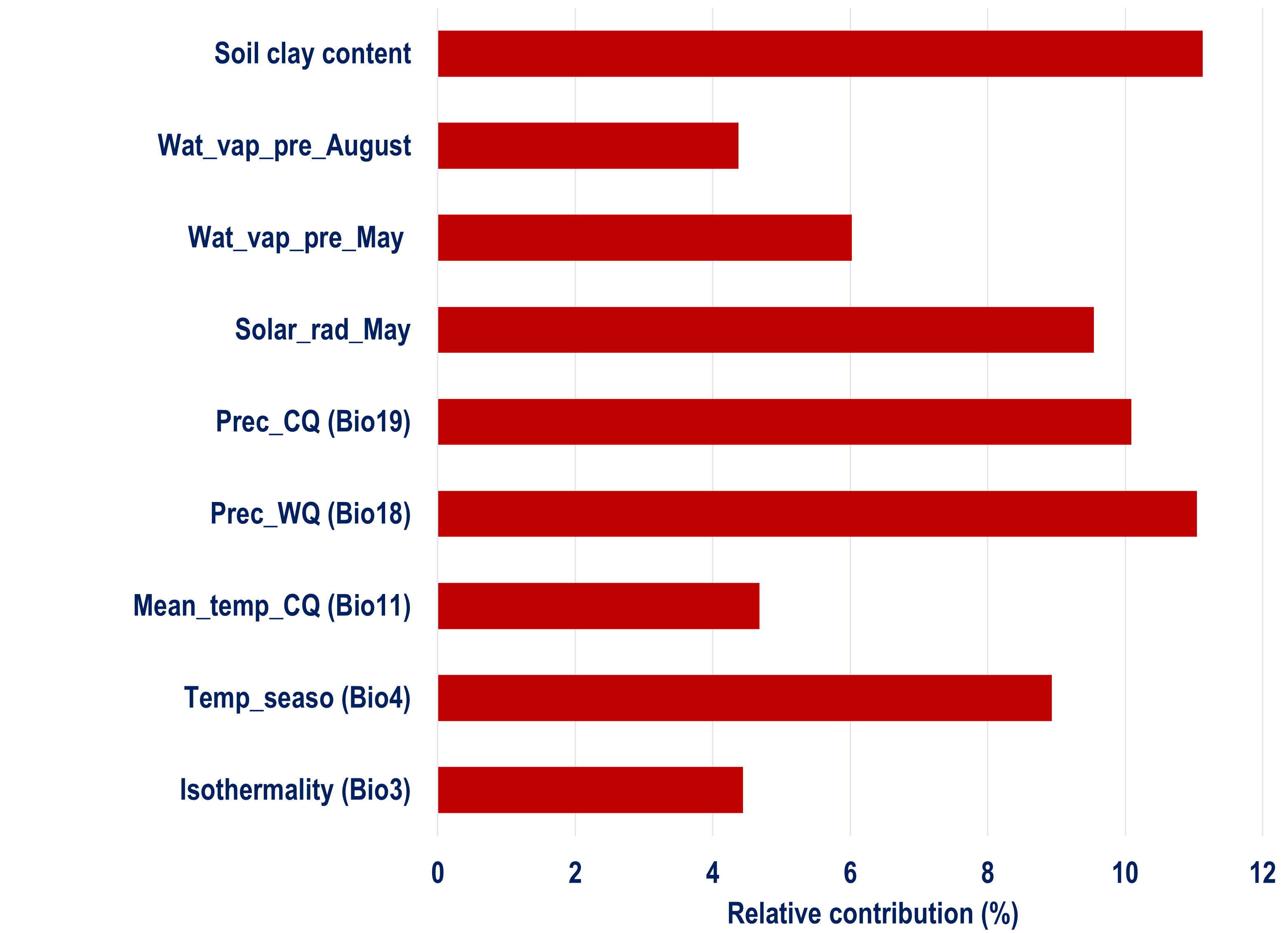

Supplement: Supplementary file 9 [file Image_4.JPEG]

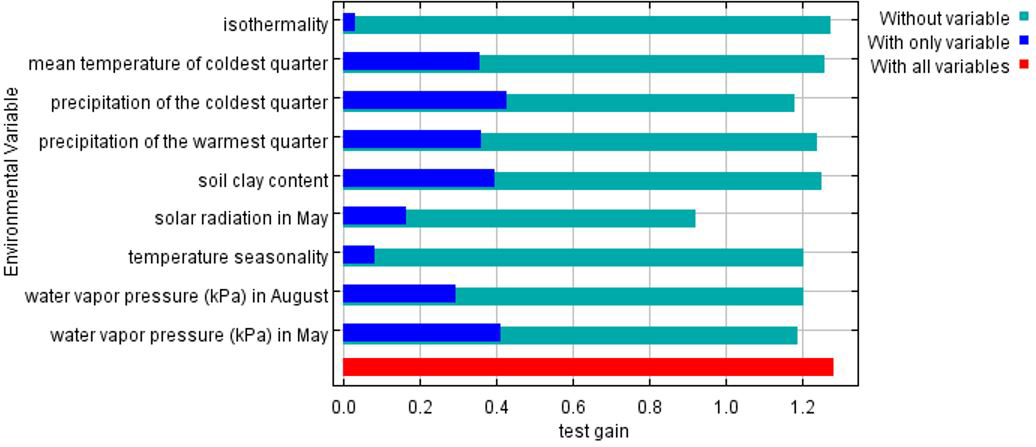

Supplement: Supplementary file 10 [file Image_5.JPEG]

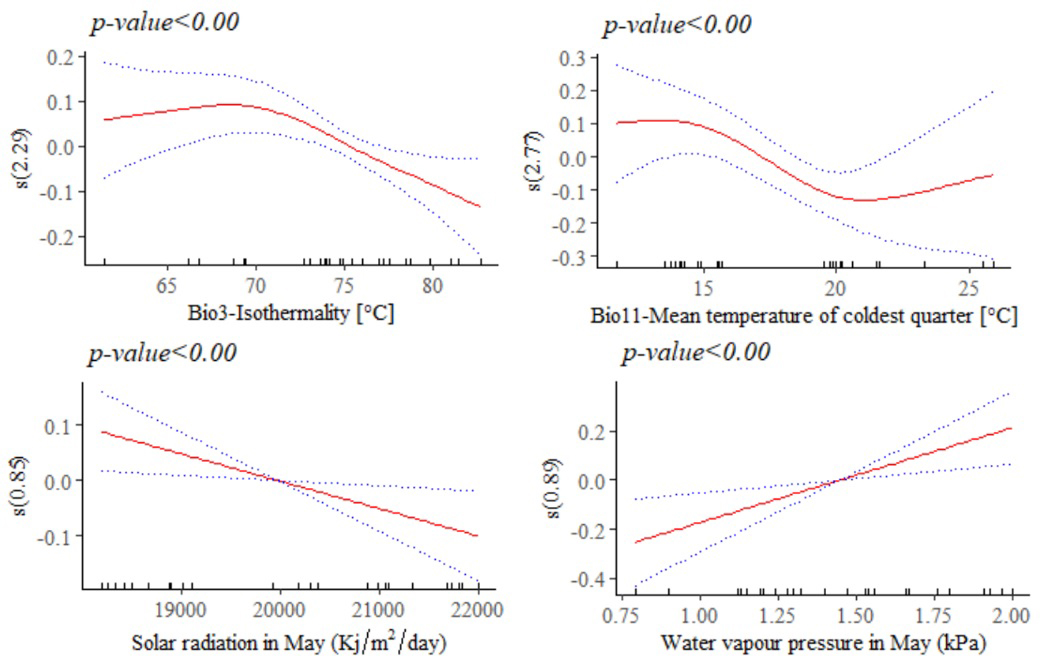

Supplement: Supplementary file 11 [file Image_6.JPEG]

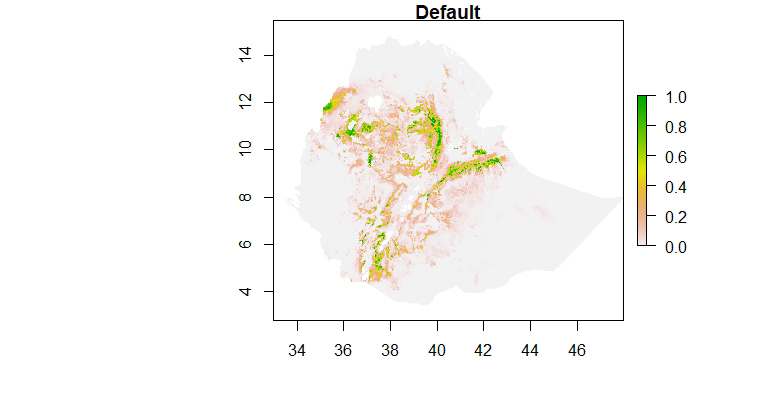

Supplement: Supplementary file 12 [file Image_7.JPEG]

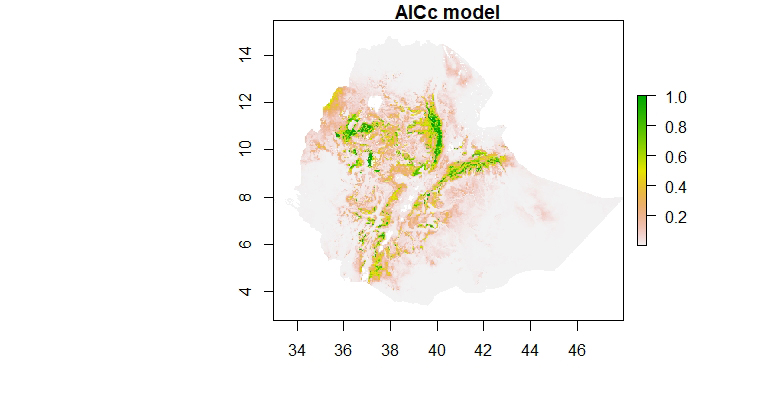

Supplement: Supplementary file 13 [file Image_8.JPEG]

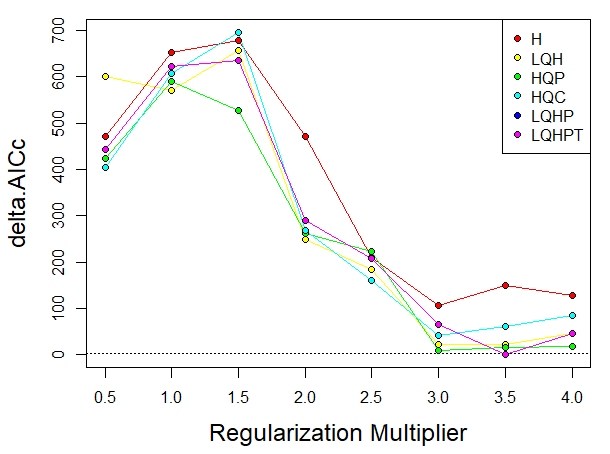

Supplement: Supplementary file 14 [file Image_9.TIFF]

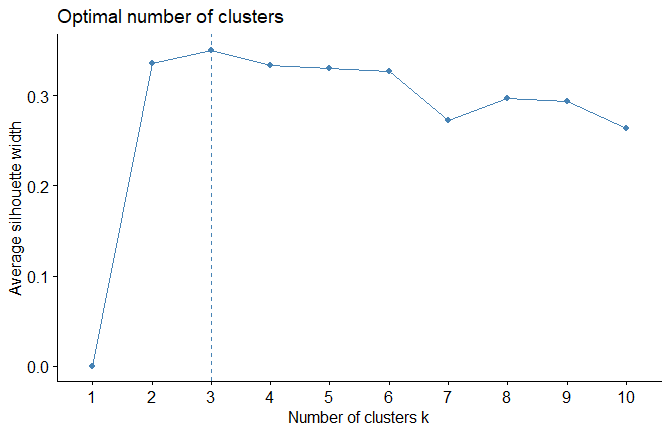

Supplement: Supplementary file 15 [file Image_10.TIFF]

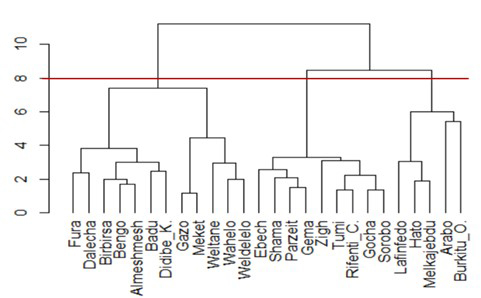

Supplement: Supplementary file 16 [file Image_11.JPEG]

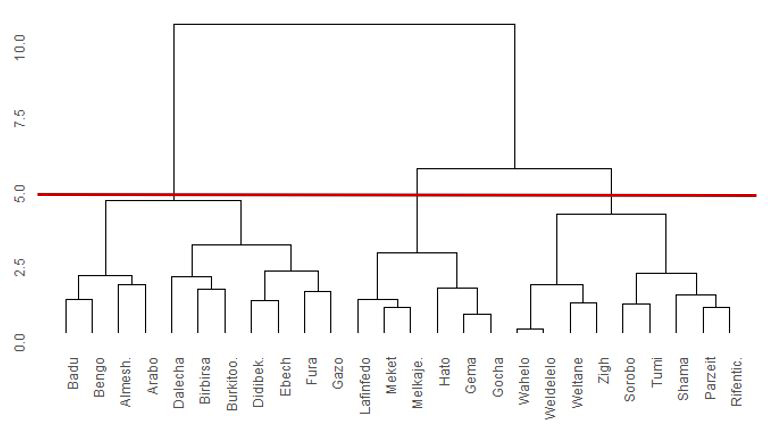

Supplement: Supplementary file 17 [file Image_12.JPEG]

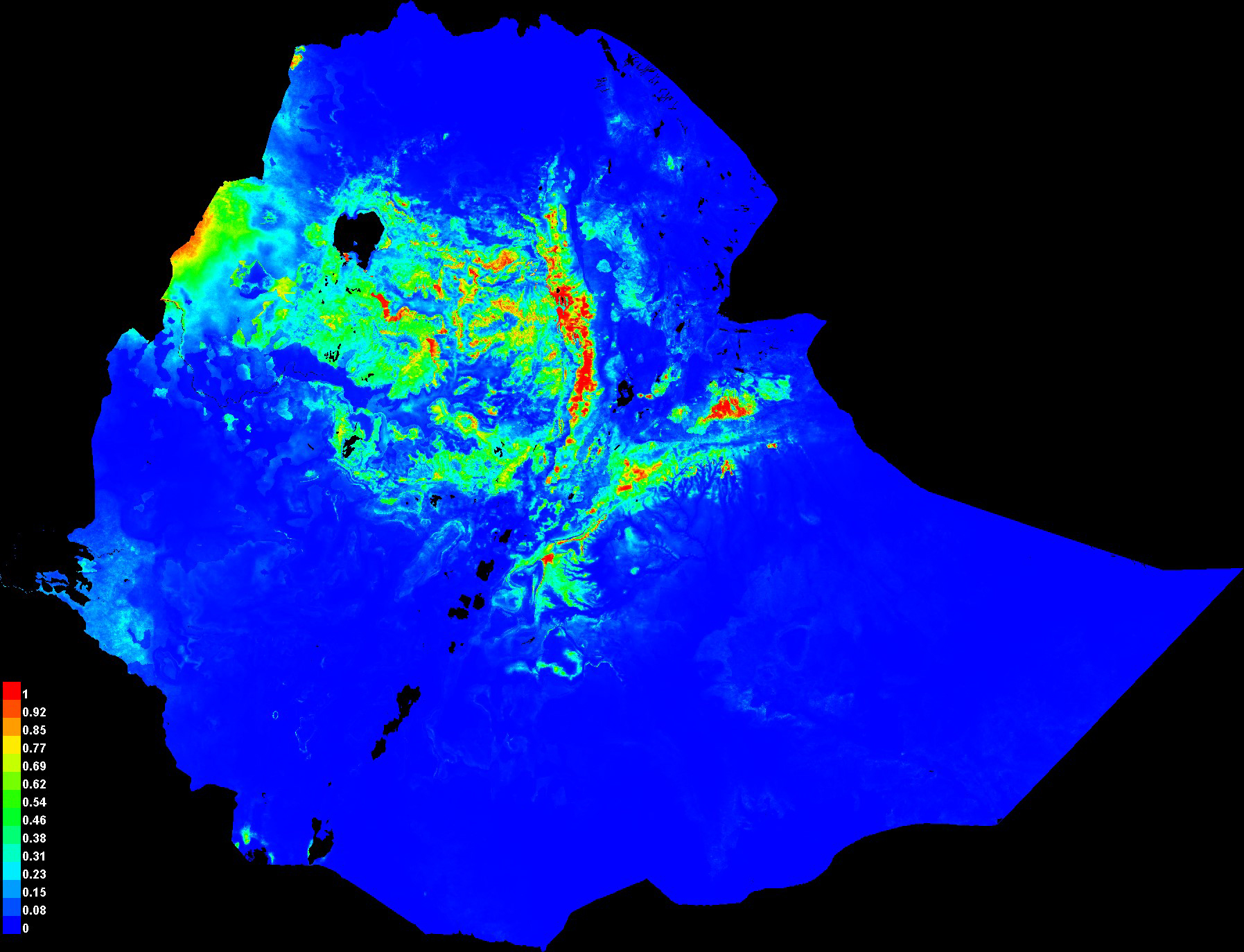

Supplement: Supplementary file 18 [file Image_13.JPEG]

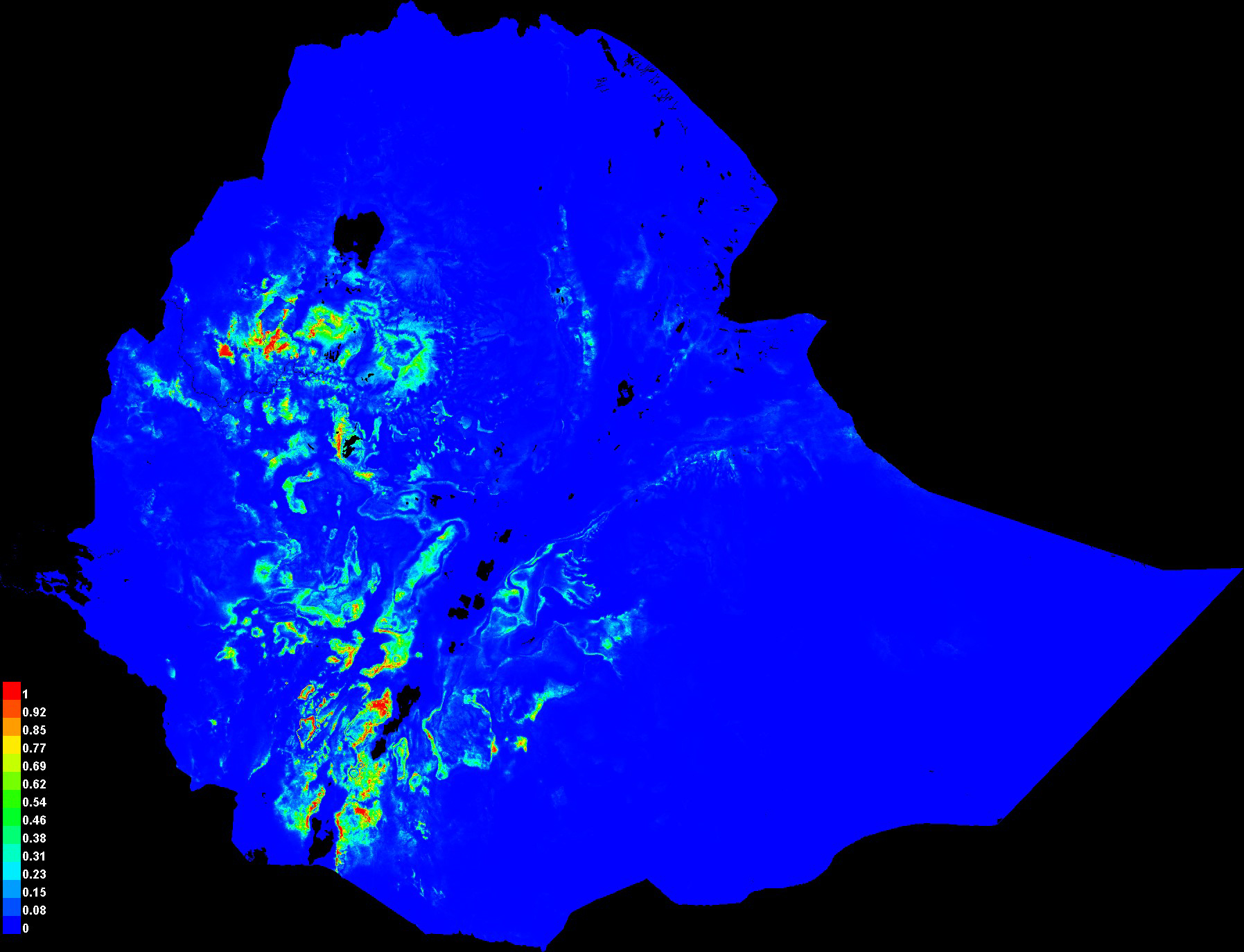

Supplement: Supplementary file 19 [file Image_14.JPEG]

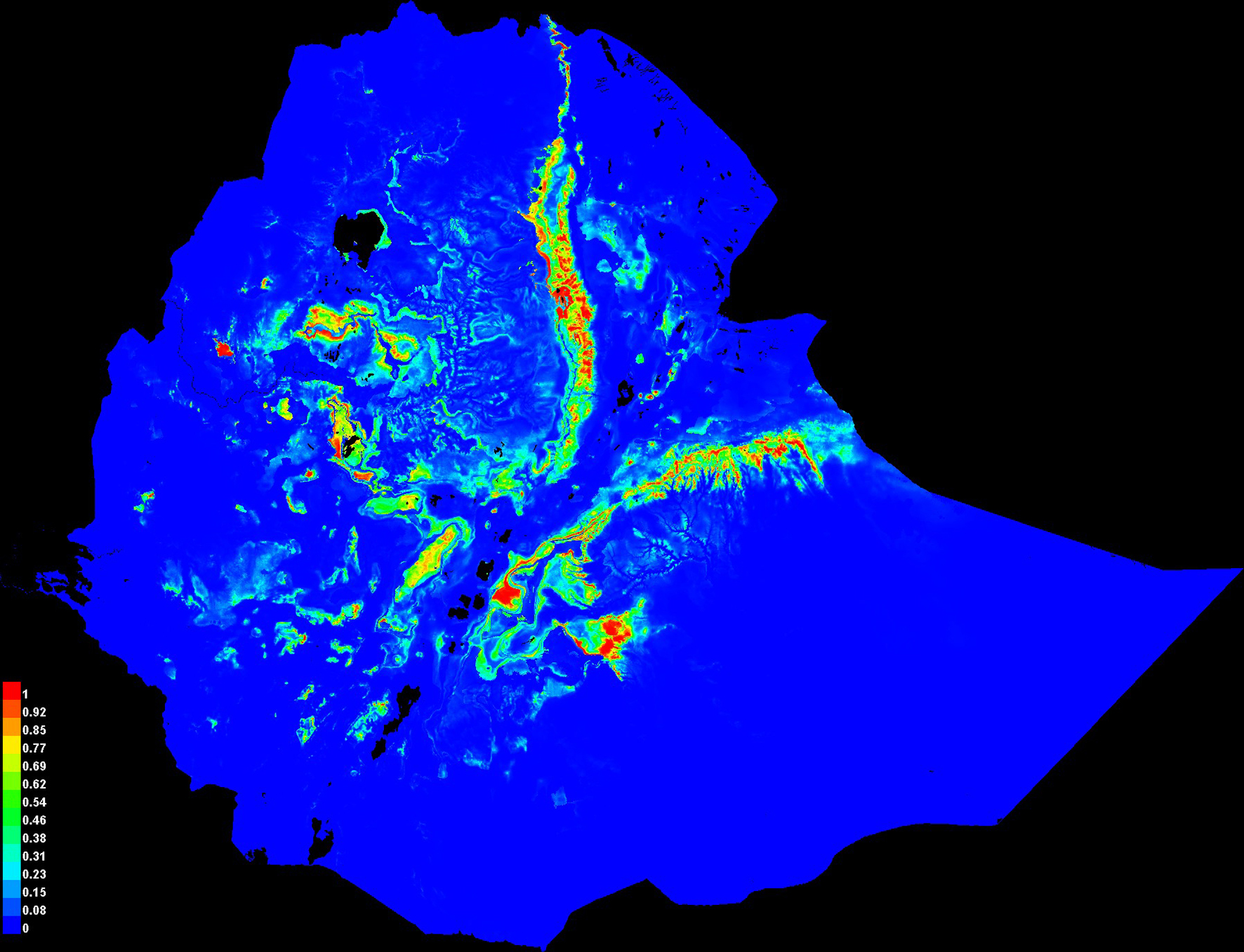

Supplement: Supplementary file 20 [file Image_15.JPEG]
